# A Human‐Specific De Novo Gene Promotes Cortical Expansion and Folding

**DOI:** 10.1002/advs.202204140

**Published:** 2023-01-13

**Authors:** Jianhuan Qi, Fan Mo, Ni A. An, Tingwei Mi, Jiaxin Wang, Jun‐Tian Qi, Xiangshang Li, Boya Zhang, Longkuo Xia, Yingfei Lu, Gaoying Sun, Xinyue Wang, Chuan‐Yun Li, Baoyang Hu

**Affiliations:** ^1^ State Key Laboratory of Stem Cell and Reproductive Biology Institute of Zoology Chinese Academy of Sciences Beijing 100101 China; ^2^ Savaid Medical School University of Chinese Academy of Sciences Beijing 100049 China; ^3^ Laboratory of Bioinformatics and Genomic Medicine Institute of Molecular Medicine College of Future Technology Peking University Beijing 100871 China; ^4^ Institute for Stem Cell and Regeneration Chinese Academy of Sciences Beijing 100101 China; ^5^ Beijing Institute for Stem Cell and Regenerative Medicine Beijing 100101 China

**Keywords:** cortical development, de novo gene, neural progenitors, proliferation

## Abstract

Newly originated de novo genes have been linked to the formation and function of the human brain. However, how a specific gene originates from ancestral noncoding DNAs and becomes involved in the preexisting network for functional outcomes remains elusive. Here, a human‐specific de novo gene, *SP0535*, is identified that is preferentially expressed in the ventricular zone of the human fetal brain and plays an important role in cortical development and function. In human embryonic stem cell‐derived cortical organoids, knockout of *SP0535* compromises their growth and neurogenesis. In *SP0535* transgenic (TG) mice, expression of *SP0535* induces fetal cortex expansion and sulci and gyri‐like structure formation. The progenitors and neurons in the *SP0535* TG mouse cortex tend to proliferate and differentiate in ways that are unique to humans. *SP0535* TG adult mice also exhibit improved cognitive ability and working memory. Mechanistically, SP0535 interacts with the membrane protein Na^+^/K^+^ ATPase subunit alpha‐1 (ATP1A1) and releases Src from the ATP1A1‐Src complex, allowing increased level of Src phosphorylation that promotes cell proliferation. Thus, *SP0535* is the first proven human‐specific de novo gene that promotes cortical expansion and folding, and can function through incorporating into an existing conserved molecular network.

## Graphical abstract

Herein, the first complete study of biological function of a human‐specific de novo protein in human cortical organoids and mice is reported. The de novo gene is potentially involved in cortical enlargement and folding during development and might participate in the acquisition of high intelligence.

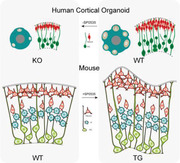

## 1 Introduction

The human neocortex has undergone substantial enlargement during evolution and has attained increased complexity for higher intellectual functions.^[^ ^1^, ^2^, ^3^ ^]^ Such cortical enlargement involves unique cortical neurogenesis, including neural progenitor amplification that is encoded by human‐specific genetic elements.^[^ ^4^, ^5^, ^6^, ^7^, ^8^, ^9^, ^10^, ^11^ ^]^ On the one hand, amending conserved genes can shape human uniqueness in cortical expansion;^[^ ^2^, ^12^, ^13^, ^14^, ^15^ ^]^ on the other hand, new genes recently evolved in humans could also be involved in shaping the human brain, which may further contribute to the advanced human intelligence.^[^ ^16^, ^17^, ^18^, ^19^, ^20^ ^]^ New genes could emerge from ancestral genes via gene duplication or originate de novo from noncoding regions.^[^ ^21^ ^]^ Pilot studies have revealed that new genes originating through gene duplication can affect cortical expansion and folding, such as the human‐specific genes *ARHGAP11B* and *NOTCH2NL*, the hominoid‐specific gene *TBC1D3*, and the primate‐specific gene *TMEM14B*, which are all preferentially expressed in the progenitors of the fetal neocortex and are linked to cortical expansion and folding.^[^ ^11^, ^22^, ^23^, ^24^, ^25^ ^]^ However, whether new genes originating de novo from noncoding DNA regions (or de novo genes) could similarly contribute to human uniqueness in brain development remains to be addressed.

Recently, we have identified a list of human‐specific, selectively constrained de novo genes in humans,^[^ ^26^ ^]^ indicating that some of them should have already been fixed with adaptive functions specifically in humans. A large portion of these genes are primarily expressed in the brain,^[^ ^26^, ^27^ ^]^ particularly in the human fetal brain^[^ ^28^ ^]^ (Figure S1b, Supporting Information). Thus, it is plausible that these new genes might play adaptive roles during human brain development. Since recent single‐cell transcriptome studies have proposed cross‐species differences in cell types in primate brains,^[^ ^29^, ^30^ ^]^ it is interesting to investigate whether these de novo genes could also contribute to cell‐type specification and further to human uniqueness in brain development. Although we and others have linked some of these new genes to human brain development and functions,^[^ ^28^, ^31^, ^32^ ^]^ no specific functions have been assigned, and the mechanisms of how de novo genes influence human brain structures remain to be addressed. Considering that these new genes have no homology with other known genes, it is quite difficult to clarify their biological functions, especially how they are incorporated into preexisting eukaryotic cellular networks. Computational tools such as Alphafold2 could predict the structures and features of new proteins; these tools are nevertheless largely based on sequence similarity to known genes and thus could provide very few informative predictions for these de novo genes. Addressing their functional implications in higher‐order functions of the human brain is another challenge. Recently, human stem cell‐derived, 3D multicellular in vitro tissue constructs (organoids) have provided a practical model to study the functions of these de novo genes. Thus, human cortical organoids together with transgenic (TG) animal models would jointly pinpoint the functions of de novo genes.

Here, we report the first case of a human‐specific de novo gene with definite functions in human brain development, particularly in cortical enlargement and folding formation that characterize the human brain. We also intend to in‐depth clarify how this new gene is involved in these phenotypes and contributes to the cognitive ability and working memory of humans.

## 2 Results

### 2.1 *SP0535* Is a Human‐Specific Protein‐Coding Gene with De Novo Origin

In a joint work of this study, using a computational pipeline for ab initio identification and meta‐analysis of de novo genes of the hominoid lineage, we identified a list of 45 human‐specific de novo genes (Table S8, Supporting Information). To further clarify the contributions of these new genes to human uniqueness in brain development, we focused on genes with relatively higher expression in brain tissues and those with differential expression in fetal brain development (Figure S1b, Supporting Information). According to these criteria, a human‐specific gene at the *ENST00000370535* locus (or *SP0535*, Figure S1a and Table S1, Supporting Information) was selected, representing the candidate with high priority for adaptive roles in human brain development (Figure S1b, Supporting Information).^[^ ^26^ ^]^

**Figure 1:**
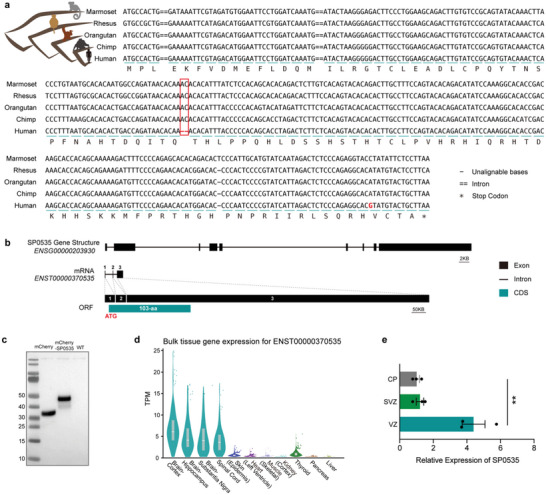
*SP0535* is a human‐specific gene with enriched expression in the cerebral cortex. a) The DNA and amino acid sequence of the CDS locus in human *SP0535*, with cross‐species alignment of orthologous genomic sequences in four other primates. A two‐base deletion (red frame) creates an ORF encoding 103 amino acids. The segregating sites are highlighted in red. b) Gene structure of *ENST00000370535*, a human‐specific de novo protein‐coding gene, was previously reported.^[^ ^27^ ^]^ c) Western blotting showing the expression of SP0535 constructs in HEK293T cells. Lanes from left to right are as follows: markers, cells transfected with mCherry (mCherry), cells transfected with the mCherry‐SP0535 fusion protein (mCherry‐SP0535), and wild‐type (WT) cells. d) Violin plot showing the expression of *SP0535* in multiple human tissues. The sample size of each tissue is as follows (from left to right): 255, 197, 139, 159, 604, 432, 803, 85, 653, 328, and 226. e) RT‒qPCR showing the relative expression of *SP0535* in the human fetal cortex (*n* = 3 each group). Data are presented as the mean ± SEM (^**^ *p* < 0.01, unpaired two‐tailed Student's *t*‐test).

Compared to that of the marmoset, rhesus macaque, orangutan, and chimpanzee, the human lineage harbors segregating sites of one single‐base mutation and a two‐nucleotide deletion in the *ENST00000370535* locus (**Figure** **1**a). Notably, the two‐nucleotide deletion specifically occurring in humans allows escape of the downstream stop codon in the orthologous sequences in out‐group species (Figure 1a). The translation of a 312‐nucleotide open reading frame (ORF) could putatively encode a 103‐amino acid protein (Figure 1b and Figure S1a, Supporting Information). Public Ribo‐seq data and large‐scale mass spectrometry data also supplement evidence regarding the transcriptional and translational expression of *SP0535* in humans (Figure S1d, Supporting Information).^[^ ^27^, ^33^ ^]^

To verify whether the de novo coding sequence (CDS) produces a complete protein, we linked the CDS of *SP0535* to the N‐terminus of mCherry, obtaining a mCherry‐SP0535 vector that can produce an mCherry‐tagged SP0535 fusion protein upon transfected into HEK293T cells (schematic diagram of Figure S1c, Supporting Information). Western blots indicated that the mass of the fusion protein increased by 12 kDa (total 44 kDa) compared to that of mCherry (32 kDa), consistent with the length of the SP0535 CDS (Figure 1c and Figure S1c, Supporting Information). As the first step to clarify the functions of this newly originated, human‐specific gene, we investigated its expression in tissues derived from the ectoderm, mesoderm, and endoderm (see Experimental Section). This gene is primarily expressed in brain tissues, especially the cerebral cortex (Figure 1d). mRNA of SP0535 in the cerebral cortex exhibits a gradient of high expression in the ventricular zone (VZ) and less intensive expression in the subventricular zone (SVZ) and cortical plate (CP) (Figure 1e), indicating a role of this new gene in human neocortex development.

### 2.2 *SP0535* Regulates Neural Development in hESC‐Derived Cortical Organoids

**Figure 2:**
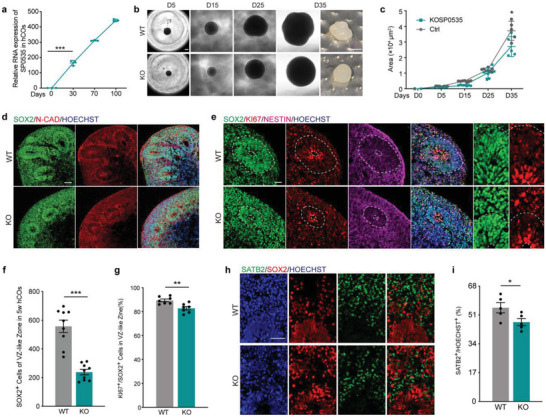
Deletion of *SP0535* impairs neural development in hESC‐derived cortical organoids. a) Relative expression of *SP0535* in human cortical organoids at 0, 30, 70, and 100 days of differentiation (*n* = 3 each group). b) Representative images of WT and *SP0535* KO organoids on days 5, 15, 25, and 35 of differentiation. Scale bar = 200 µm (left) and 500 µm (right). c) *SP0535* KO organoids gradually became smaller than WT organoids from day 5 of differentiation (*n* = 7 each group). d,e) Staining for SOX2, N‐cadherin (N‐CAD), KI67, and nestin in hCOs after 5 weeks. The dashed lines delimit the VZ. Scale bar, 100 µm (d) and 50 µm (e). f,g) Quantification of SOX2^+^ and KI67^+^ cells in hCOs after 5 weeks (*n* = 9 each group in [f] and *n* = 7 each group in [g]). h) Staining for SATB2 and SOX2 in hCOs after 10 weeks of differentiation. Scale bar = 50 µm. i) Quantification of the percentage of SATB2^+^ cells in hCOs after 10 weeks (*n* = 5 each group). Data are presented as the mean ± SEM (^*^ *p* < 0.05, ^**^ *p* < 0.01, and ^***^ *p* < 0.001, unpaired two‐tailed Student's *t*‐test).

To further investigate whether SP0535 could induce human‐specific features in the cortex, we generated human embryonic stem cell (hESC)‐derived cortical organoids (hCOs) (Figure S2a, Supporting Information). The expression of *SP0535*, triggered by neural differentiation, started to increase on day 30 of differentiation and continuously increased thereafter (**Figure** **2**a and Figure S2b, Supporting Information). An SP0535 knockout (KO) hESC line was also generated for loss‐of‐function assays (Figure S2c, Supporting Information). Although SP0535 KO does not affect the pluripotency and proliferation of hESCs based on OCT4 immunostaining and the EdU assay (Figure S2d–f, Supporting Information), upon differentiation, the hCOs generated from the SP0535 KO hESC line are significantly smaller, especially after 5 weeks of differentiation (Figure 2b,c). The outer surface of these cortical organoids tends to be smoother than those of the WT hCOs (Figure 2b). KO of SP0535 did not affect the overall cell density of hCOs (Figure S2h, Supporting Information), but it significantly decreased the number of individual cortex‐like structures within the hCOs (Figure 2d and Figure S2g, Supporting Information). Consistently, either the number of SOX2^+^ radial glia (RG) cells or the proportion of PAX6^+^ apical progenitors significantly decreased in these organoids (Figure 2e,f and Figure S2i,j, Supporting Information). Of note, the number of KI67‐ and SOX2‐coexpressing RG cells in cell cycle decreased significantly in the *SP0535* KO hCOs at 5 weeks of differentiation (Figure 2e,g). Such alterations in deep‐layer neurons are also similar in 10‐week‐old *SP0535* KO hCOs (Figure 2h,i). The expression profile of other markers, such as *SOX2*, *PAX6*, *KI67*, *SATB2*, and *DCX* (Figure S2k–o, Supporting Information), further demonstrates that KO of SP0535 impairs neurogenesis in hCOs.

### 2.3 SP0535 Induces Cortical Enlargement and Formation of Sulci‐ and Gyri‐Like Structures in Mice

**Figure 3:**
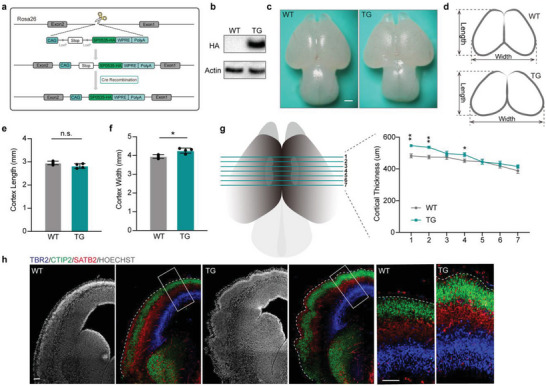
*SP0535* promotes cortical expansion and sulci and gyri formation in mice. a) Schematic design of the transgenic mice. The *SP0535* coding sequence was subcloned with an HA‐tag to facilitate the detection of SP0535 expression. SP0535 is expressed in the whole body using this system. b) Western blots for HA‐tagged SP0535 with actin as an internal control. c) Representative images of the E15.5 brains of WT and *SP0535‐TG* mice. Scale bar = 500 µm. d) Schematic diagram showing the length and width of the brain cortex. e,f) Length (e) and width (f) of the cortex of the E15.5 WT and TG mice (*n* = 4 each group). g) Cortical thickness of E15.5 WT and TG mice at the indicated positions (see cartoon, *n* = 3 each group). h) Immunostaining of the IP marker (TBR2) and layer‐specific markers (CTIP2 and SATB2) in WT and *SP0535‐TG* mice at E15.5, showing gyrus and sulcus structures induced by SP0535. Dashed lines delimit the cortical surface. Scale bar = 100 µm. Data are presented as the mean ± SEM (^*^ *p* < 0.05 and ^**^ *p* < 0.01, unpaired two‐tailed Student's *t*‐test).

To investigate the function of SP0535 in cortical development, we further generated TG mice in which human *SP0535* driven by a DPPA3‐Cre‐based system was inserted into the mouse genome (**Figure** **3**a). We inserted an HA‐tag at the N‐terminus of SP0535 for subsequent verification of its expression (Figure 3b). As *SP0535* is highly expressed in the VZ region of the human fetal cerebral cortex, we focused on the alteration of the mouse cortex at E15.5, a vital period for amplifying neural progenitor cells (NPCs).^[^ ^7^ ^]^ Although the TG mice did not show a significant difference in brain weight (Figure S3a,b, Supporting Information), the cortices of some TG mice at E15.5 become larger, and certain stratifications appear thickened (Figure 3c–f). To verify this, we divided the cortex into seven portions (schematic diagram of Figure 3g) from the rostral to caudal end and measured the cortical layers on the coronal plane of each portion (Figure S3c, Supporting Information). Interestingly, at positions 1, 2, and 4, especially positions 1 and 2, which generally represent the prefrontal lobe, the cortex becomes considerably thickened (Figure 3g). In addition, the E15.5 TG mice exhibit cortical gyrification, as is evident in immunostaining against TBR2 (also known as EOMES), CTIP2 (also known as BCL11B), and SATB2 (Figure 3h and Figure S3d, Supporting Information). In all TG mice with thickened cortices, the penetrance of folding at E15.5 was 37.5% (6 out of 16 mice). Folding does not appear with clear left‐ or right‐sided preference, but all in the developing prefrontal cortex. We employed a ratio of outer and inner contours at the same straight‐line distance as the local gyrification index (GI) to quantify the extent of folding. The TG mice exhibited significantly increased local GI (Figure S3e, Supporting Information). Overall, the neocortex of *SP0535*‐TG mice undergoes significant morphological and structural alterations that are related to cortical folding at E15.5.

### 2.4 *SP0535* Promotes the Amplification of Cortical Progenitors in *SP0535‐*TG Mice

**Figure 4:**
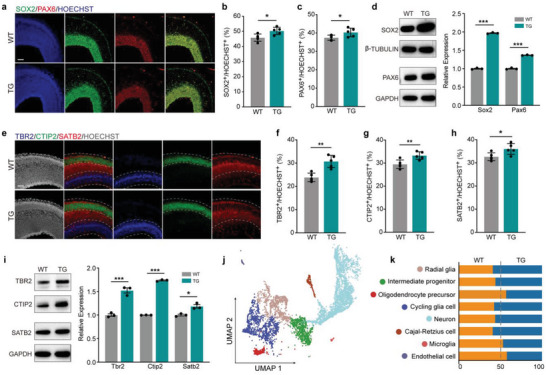
SP0535 promotes the expansion of neural progenitors and neurons in *SP0535‐TG* mice. a) Immunostaining for SOX2 and PAX6 in E15.5 WT and TG mice. Scale bar, 100 µm. b,c) Quantification of SOX2^+^ (b) and PAX6^+^ (c) cells in E15.5 cortices (*n* = 5 each group). d) Western blots and quantification of SOX2 and PAX6 expression in E15.5 mouse cortices (*n* = 3 each group). e) Immunostaining for TBR2, CTIP2, and SATB2 in WT and TG mouse cortices at E15.5. Scale bar = 100 µm. f–h) Quantification of TBR2^+^ (f), CTIP2^+^ (g), and SATB2^+^ (h) cells in E15.5 cortices (*n* = 5 each group). i) Western blots and quantification of TBR2, CTIP2, and SATB2 expression in E15.5 cortices (*n* = 3 each group). j) UMAP visualization of scRNA‐seq data of E15.5 WT and TG cortices. k) Proportion of cell types in WT and TG cortices at E15.5. Data are presented as the mean ± SEM (^*^ *p* < 0.05, ^**^ *p* < 0.01, and ^***^ *p* < 0.001, unpaired two‐tailed Student's *t*‐test).

RG cells are a key NPC type that generate IP cells and subsequent neurons for cortical expansion and neural development.^[^ ^8^, ^34^ ^]^ Immunostaining and Western blotting data showed that *SP0535‐TG* mice generate more RG cells, exclusively in the VZ at E15.5, the critical stage when mice undergo intensive neurogenesis (**Figure** **4**a–d and Figure S4c, Supporting Information). Accordingly, the expression of *Sox2* and *Pax6* is also upregulated (Figure S4d,e, Supporting Information). The *SP0535‐TG* mice also generate more IP cells as well as deeper and upper layer neurons, characterized by the expression of TBR2, CTIP2, and SATB2, respectively (Figure 4e–i and Figure S4f–h, Supporting Information). In addition, we found a significantly increased number of TUJ1^+^ immature neurons and upregulated *DCX* expression in the *SP0535‐TG* cortices (Figure S4a,b,i, Supporting Information), supporting the idea that *SP0535* induces the generation of more newborn neurons.

To further investigate how SP0535 affects the cell composition in the mouse cortex, we performed single‐cell RNA sequencing (scRNA‐seq) based on 10× Genomics. In total, 15 021 cells from the E15.5 brains of WT and TG mice were dissociated and collected. Uniform manifold approximation and projection for dimension reduction (UMAP) was then performed on cortical cells of both genotypes (Figure 4j) according to published marker genes.^[^ ^35^, ^36^, ^37^ ^]^ Several separate cell clusters (Figure 4k) were identified, including RG (*Sox2, Slc1a3*, *Ptprz1*), intermediate progenitors (*Eomes*, *Neurog2*), cycling glial cells (*Ki67*, *Sox2*, *Nkx2‐1*), Cajal‐Retzius cells (*Reln*), neurons (*Satb2*, *Bcl11b*, *Foxp2*, *Cux2*), oligodendrocyte precursors (*Adgrv1*), microglia (*Trem2*), and endothelial cells (*Igfbp3*, *Mcam*). The distributions of the representative cell types are shown in Figure S4k–r, Supporting Information. According to the cell types defined by the transcriptome, although no new cell type as a group appears in cortices of the *SP0535‐TG* mice (Figure S4j, Supporting Information), the proportion of progenitor cells, especially RG cells, is increased in TG mice, which eventually causes an expansion of the neuronal population (Figure 4k). These data collectively indicate that *SP0535* affects cell proliferation and population expansion during cortical development.

### 2.5 SP0535 Improves Cognitive Ability and Working Memory in SP0535‐TG Adult Mice

**Figure 5:**
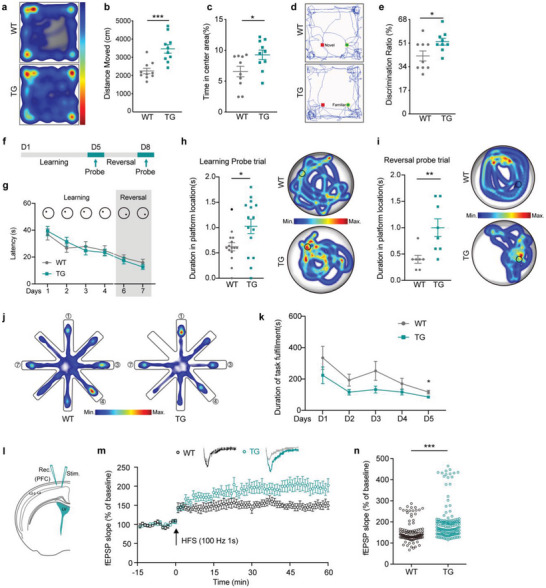
*SP0535*‐TG improves cognitive ability and working memory in adult mice. a) Representative tracing path of WT and *SP0535*‐TG mice in the open field test. b,c) Total moving distance (b) and time spent in the center area (c) of mice in the open field (*n* = 10 each group). d) Representative tracing path of WT and *SP0535‐TG* mice in the novel object recognition (NOR) test. Green circles and red squares represent familiar and novel objects, respectively. e) Discrimination ratio of WT and *SP0535‐TG* mice in the NOR test. The discrimination ratio was calculated by dividing the time required for touching the novel object by the time required for touching two objects (*n* = 9 each group). f) Schematic showing the experimental procedure of the MWM. g) Latency to platform of WT and *SP0535‐TG* mice in the MWM test. h) Time spent in the platform location when the platform was hidden after 4 days of training (*n* = 15 each group). Representative moving paths are shown on the right. i) Time spent in the reversal platform location when the platform was hidden after 2 days of reversal training (*n* ^WT^ = 7 and *n* ^TG^ = 8). Representative moving paths are shown on the right. j) Representative tracing path of WT and *SP0535‐TG* mice in the 8‐arm maze test. Arms 1, 3, 4, and 7 were the right arms with reward. k) Latency to the target arm of WT and *SP0535‐TG* mice in the 8‐arm maze test. l) Schematic showing the electrophysiological experiments in WT and *SP0535‐TG* mice. fEPSPs were recorded at L2–3 after stimulating L4 in the prefrontal cortex. m) Electrical stimulation in the cortex (L4) generated fEPSPs, as recorded using a glass electrode placed in L2 + 3. Stable measurements of fEPSP amplitude were followed by high‐frequency stimulation (HFS). Representative traces of fEPSPs 5 min before (gray line) and 55 min after (black or blue line) HFS (100 Hz, 1 s, top). A summarized plot of the fEPSP slope shows the induction of long‐term potentiation (LTP) by HFS that lasts for 60 min (bottom). n) Bar histogram showing quantified data within the last 5 min of the LTP recording. Data are presented as the mean ± SEM (^*^ *p* < 0.05, ^**^ *p* < 0.01, and ^***^ *p* < 0.001, unpaired two‐tailed Student's *t*‐test).

Since SP0535‐TG mice exhibit an expanded cortex and sulci and gyri‐like structure in the prefrontal cortex at E15.5, a key region implicated in modulating cognitive ability and memory, we next investigated whether the ectopic expression of SP0535 induces behavioral alterations in adult mice. We performed behavioral tests that indicate cognitive capability (novel object cognition), spatial learning and memory (Morris water maze [MWM]), and working memory (eight‐arm radial maze). The *SP0535*‐TG mice tended to travel longer distances in the open‐field test, including the whole ground and the central area (**Figure** **5**a–c), indicating more active voluntary exploration. The results of the RotaRod test rule out possible differences in motor ability between the TG and WT mice (Figure S5a, Supporting Information). In the novel object recognition test, TG mice exhibited significantly increased cognitive ability for novel objects compared with the WT mice (Figure 5d,e). To further assess the contributions of *SP0535‐*TG to learning and memory abilities in mice, we designed regular and reversal training paradigms in the MWM (Figure 5f,g). After normal training trials, although the TG mice did not exhibit improvement in the learning curve, they stayed longer in the platform location, indicating improved memory (Figure 5h). In the subsequent reversal training trials, the TG mice exhibited memory of the new platform location (Figure 5i), indicating that SP0535 improves the working memory of mice. In the eight‐arm radial maze assay that directly targets the degrees of working memory in mice, as expected, the TG mice generally spent less time completing the task (see details in the Experimental Section), which means they entered the wrong arm less frequently than the WT mice (Figure 5j,k), demonstrating a significant improvement in working memory. In the three‐chamber social test, the TG mice did not show significant differences in social patterns (Figure S5b,c, Supporting Information). Collectively, these data provide evidence that ectopically expressing SP0535 significantly improves working memory in mice.

To further verify the findings in animal behavioral experiments, we performed electrophysiological recording of the prefrontal cortices of the TG and WT mice (Figure 5l). Briefly, we performed patch‐clamp experiments on brain slices from 6 WT and 6 TG mice that were used in the behavioral experiments, using 11 and 14 slices, respectively. After high‐frequency stimulation (HFS), we found that the field excitatory postsynaptic potential (fEPSP) of the TG prefrontal cortex was significantly higher than that of the WT (Figure 5m,n), indicating the generation of a more potent long‐term potentiation (LTP), the basis of learning and memory, in SP0535‐TG mice.

Next, we examined the thickness and total number of neurons in the prefrontal cortex and the number of neurons in layers 2, 3, and 4 (Figure S5d–i, Supporting Information). Although we did not observe significant differences in neuron numbers (Figure S5f,h,i, Supporting Information), certain regions of the deep layer exhibited reorganization and aggregation of deep‐layer neurons (Figure S5g,j,k, Supporting Information). The penetrance is around 37.5% (three out of eight, Figure S5g, Supporting Information). As neither cell amount nor cell type composition exhibits differences in other brain regions between the two groups (Figure S5l,m, Supporting Information), the behavioral improvements detected were likely primarily caused by alterations of the cortical region.

### 2.6 SP0535 Incorporates into the Preexisting Network through ATP1A1

**Figure 6:**
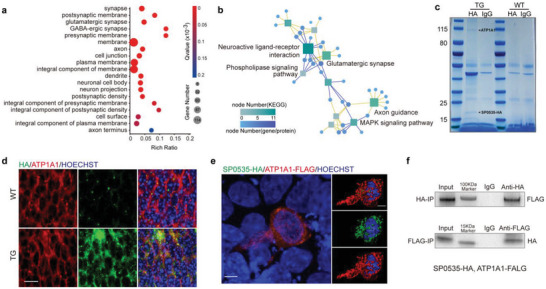
SP0535 interacts with ATP1A1. a) Enriched GO terms for upregulated genes in radial glia of *SP0535‐TG* mice. The sizes of the bubbles indicate the number of differentially expressed genes associated with a given GO term. The *Q* values for each term are shown according to the color scale. b) Enriched KEGG pathways for differentially expressed genes in radial glia of *SP0535‐TG* mice. The numbers of upregulated genes associated with each pathway are shown according to the size of the squares and the color scale. c) Immunoprecipitation of SP0535‐HA. d) Colocalization of SP0535‐HA and ATP1A1 in the cortices of WT and *SP0535‐TG* mice at E15.5. Scale bar = 10 µm (left) and 5 µm (right). e) Coimmunostaining of HA‐tagged SP0535 and FLAG‐tagged ATP1A1 in HEK293T cells. Scale bar = 5 µm. f) Coimmunoprecipitation using an anti‐HA antibody (top panel) and anti‐FLAG antibody (bottom panel).

To probe how SP0535 promotes cortical development and cognition in mice, we performed functional enrichment analyses with Gene Ontology (GO) terms and Kyoto Encyclopedia of Genes and Genomes (KEGG) pathways for differentially expressed genes between *SP0535‐TG* mice and WT mice at E15.5 in pseudobulks of radial glia from scRNA‐seq (**Figure** **6**a,b and Figure S6a, Supporting Information). The differentially expressed genes in radial glia of *SP0535‐TG* mice were enriched in GO terms of “plasma membrane,” “integral component of the membrane,” and “synaptic activity” (Figure 6a), as well as KEGG pathways of energy‐related cellular pathways, suggesting the possible involvement of *SP0535* in regulating genes associated with membrane binding and transmembrane cellular transduction (Figure 6b).

To further elucidate the mechanism of how this new gene is incorporated into the preexisting cellular networks, we performed protein immunoprecipitation on E15.5 brain cortices, followed by mass spectrometry analysis to identify the interacting proteins of SP0535 (Table S6, Supporting Information). We identified a membrane‐binding protein, Na^+^/K^+^ ATPase subunit alpha‐1 (ATP1A1) interacts with SP0535 (Figure 6c and Figure S6b, Supporting Information), which is also confirmed by coimmunostaining of SP0535‐HA and ATP1A1 in the E15.5 mouse cortex (Figure 6d). To determine the localization of SP0535 within human cells, we co‐transfected HA‐tagged SP0535 and FLAG‐tagged ATP1A1 into HEK293T cells and found the colocalization of SP0535 and ATP1A1 at the plasma membrane (Figure 6e). Immunoblotting showed that SP0535‐HA and ATP1A1‐FLAG are specifically precipitated by each other (Figure 6f and Figure S6e, Supporting Information). These observations jointly suggest that SP0535 binds with ATP1A1 and thereby incorporates into the preexisting regulatory network.

### 2.7 SP0535 Prompts Apical Progenitor Proliferation through Regulating ATP1A1

**Figure 7:**
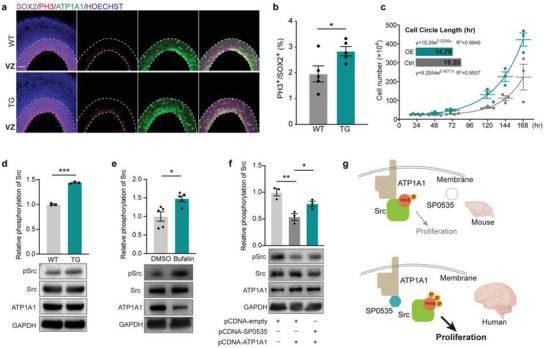
SP0535 promotes RG proliferation by perturbing the ATP1A1‐Src complex. a) Coimmunostaining of SOX2, PH3, and ATP1A1 in cortices of VZ in WT and *SP0535‐TG* mice. Scale bar = 100 µm. b) Quantification of PH3^+^ cells in Sox2^+^ cells from E15.5 cortices in WT and *SP0535*‐TG mice (*n* = 5 each group). c) Cell cycle assay in WT mice and SP0535‐overexpressing HEK293T cells. d) Western blotting and quantification of the expression of ATP1A1, Src, and phosphorylated Src (pSrc) in cortices of WT and *SP0535‐TG* mice (*n* = 3 each group). e) Western blotting showing the repressive effect of ATP1A1 on Src phosphorylation using bufalin as an inhibitor that can block ATP1A1‐Src complex formation (*n* = 5 each group). f) Western blotting showing the effect of SP0535 on Src phosphorylation. The expression of ATP1A1, Src, and pSrc in HEK293T cells was also quantified in groups transfected with empty vector, co‐transfected with ATP1A1, or co‐transfected with ATP1A1 and SP0535 (*n* = 3 each group). g) Schematic showing the function of SP0535 in regulating cell proliferation through interaction with ATP1A1. Data are presented as the mean ± SEM (^*^ *p* < 0.05, ^**^ *p* < 0.01, and ^***^ *p* < 0.001, unpaired two‐tailed Student's *t*‐test).

ATP1A1 is an *α*‐subunit isoform of Na^+^/K^+^‐ATPase, an evolutionarily conserved enzyme that catalyzes active transport of cations via hydrolysis of ATP across the cell membrane in all mammalian cells.^[^ ^38^ ^]^ Studies have shown that ATP1A1 is involved in cell proliferation in brain ventricle development, glioma stem cell proliferation, and hepatocellular carcinoma.^[^ ^39^, ^40^, ^41^ ^]^ ATP1A1 can bind with Src, a tyrosine kinase closely associated with cell proliferation and widely recognized as a proto‐oncogene.^[^ ^42^, ^43^ ^]^ Perturbation in the ATP1A1‐Src complex can alter Src phosphorylation and subsequently trigger downstream pathways. Considering the interaction of SP0535 and ATP1A1, we assumed that SP0535 may regulate the generation of cortical progenitors by modulating the ATP1A1‐Src complex. To this end, we first examined the germinal zone of the E15.5 cortical VZ and observed that ATP1A1 was enriched on the apical side of the VZ (**Figure** **7**a). Importantly, the number of PH3‐positive cells was increased considerably in TG mouse cortices (same as that observed in the EdU‐labeling assay) (Figure 7a,b and Figure S6c,d, Supporting Information). Consistently, in SP0535‐overexpressing HEK293T cells, the cell cycle also becomes shorter (Figure 7c). In the cortices of E15.5 mice, although the expression of ATP1A1 is comparable between the WT and the TG mice (Figure 7d), the level of Src phosphorylation is significantly higher in the TG mice than in the WT mice, indicating that the presence of SP0535 intervenes with the effect of ATP1A1 on Src phosphorylation (Figure 7d and Figure S6f, Supporting Information). To confirm how ATP1A1 affects the Src phosphorylation, we treated HEK293T cells with bufalin, an inhibitor that can block ATP1A1‐Src complex formation. As expected, inhibiting the ATP1A1‐Src complex formation significantly increases the level of Src phosphorylation (Figure 7e and Figure S6g, Supporting Information). Consistent with the observation in TG mice, the impaired Src phosphorylation in ATP1A1‐overexpressing HEK293T cells can be rescued by SP0535 overexpression (Figure 7f and Figure S6h, Supporting Information). These findings thus suggest that SP0535 competitively interacts with ATP1A1 and perturbs the stability of the ATP1A1‐Src complex, thereby allowing increased level of Src phosphorylation that promotes cell proliferation (Figure 7g).

## 3 Discussion

Understanding the functions of newly originated genes could provide novel insight into the genetic foundation underpinning human‐specific traits. New genes arising through gene duplication have been extensively investigated for their modulation of cortical progenitor proliferation and brain functions.^[^ ^11^, ^18^, ^19^, ^22^, ^23^, ^44^, ^45^, ^46^, ^47^ ^]^ For such new genes, the annotations of their “mother genes” can provide clues for their functional or mechanistic significance, which can also be predicted based on their sequences by computational tools such as Alphafold2. In contrast, for de novo genes, it is quite difficult to clarify their functions (Figure S7d, Supporting Information). It is also challenging to investigate how these “brand new,” de novo genes are incorporated into the preexisting molecular network. Here, we present the first case of functional study on a human‐specific protein‐coding gene with de novo origination, *SP0535*. We revealed that *SP0535* induces expansion of neural progenitors in the developing cortex, prompts the formation of sulci and gyri structures in the cortex and improves cognitive ability. We also found that SP0535 regulates cortical progenitor proliferation via direct interaction with ATP1A1, the type‐I *α* subunit of Na^+^/K^+^‐ATPase that affects Src phosphorylation.^[^ ^48^ ^]^ We thus highlight the contributions of human‐specific de novo genes to adaptive evolution in human brain development and reveal how an evolutionarily novel factor incorporates into preexisting machinery to perform important functions.

To study the function of any gene, the expression profile is informative. The in vivo expression of the SP0535 protein was supported by public large‐scale mass spectrometry data. Since various model systems were employed in this study, we carefully examined the expression pattern of SP0535 in human organoids, fetal brains, TG mice, and other cell lines involved. In human organoids, we confirmed the regional and temporal expression of this new gene and verified that the observed phenotypes were not due to off‐target effects caused by editing or by bias in cell line selection of hESCs. To further verify the expression of this new gene in humans, we attempted to generate an antibody against SP0535. Using this antibody, we validated the protein expression of SP0535 in cortical organoids grown from hESCs but not in those from SP0535‐KO hESCs (Figure S7a, Supporting Information). The findings simultaneously verified the efficiency of the KO assay. As no off‐targets were found in the gene editing system using CRISPR/Cas9, the phenotypes of SP0535‐KO organoids are unlikely caused by off‐targets (Experimental Section and Table S3, Supporting Information). The strikingly opposite alterations in the SP0535‐KO hCOs and SP0535‐TG mice further indicate that the observed phenotypes are not due to off‐target effects. Considering that the cortical organoid technique is reproducible among hES cell lines (Figure S1a, Supporting Information, and ref. [49]), and in hCOs from either hESC line H9 or H1, the expression pattern of SP0535 is consistent (Figure S1b, Supporting Information), we used H9 for the rest of the assays. Although additional investigations such as rescue assays in SP0535‐KO lines or parallel studies using different lines are ideal, the data and findings in this study are sound and representative.

The spatial expression information for the new gene is also important to deduce its functions and to facilitate the understanding of the phenotypes it induces. We explored the expression profile of *SP0535* among various cell types during human brain development through reanalyzing a public dataset of single‐cell transcriptome data at GW14. It showed that *SP0535* is broadly expressed in multiple cell types (Figure S7c, Supporting Information).^[^ ^50^ ^]^ To further investigate the spatial expression of *SP0535* in human brains, we then reanalyzed public spatial transcriptome data for the prefrontal cortex of adult humans.^[^ ^51^ ^]^ Interestingly, *SP0535* is predominantly expressed in cells of the prefrontal cortex layers 2–4 (Figure S7b, Supporting Information),^[^ ^52^ ^]^ a distribution recapitulating the phenotypes of those TG mice that exhibit enhanced cognitive capability and learning memory. Because of the lack of preferable sequences and workable protocols for in situ hybridization in human cortical organoids, a direct presentation of in situ mRNA expression is impractical. The specificity of the antibody against SP0535 also did not allow for immunofluorescence on organoids (Figure S7a, Supporting Information).

As new genes typically interact with other coevolved genetic elements to produce specific new phenotypes, it would be difficult to deduce their functions by ectopically expressing them in mice at the physiological levels of the developing human neocortex, especially for these de novo genes without homology with other known genes. As a proof‐of‐concept study to investigate how these “brand new” genes were incorporated into the preexisting molecular network, we used a strong promoter to express the full‐length CDS of *SP0535* in mice (Figure 3a,b). Of course, expression of *SP0535* in mice at the physiological level, especially using tissue‐ or cell type‐specific promoters and inducible regulatory elements, is ideal to clarify the temporospatial regulation of SP0535 in the cerebral cortex, and to exclude the possible artificial effects of ectopic expression. Nevertheless, the consistent phenotypes detected in various assays and designs jointly support the notion that the new gene promotes cortical expansion and folding.

In mouse cortical development, the stages of E12.5‐E17.5 that correspond to the most pronounced neurogenesis are widely used.^[^ ^7^, ^12^, ^16^, ^24^, ^53^, ^54^, ^55^, ^56^, ^57^, ^58^, ^59^, ^60^, ^61^, ^62^, ^63^, ^64^, ^65^, ^66^ ^]^ In our study, we assessed the expression of SP0535 in the mouse cortex at E13.5, E15.5, and E17.5 and found that SP0535 was most differentially expressed between the WT and TG mice at E15.5. Accordingly, the number of neural progenitors is also increased in TG mice. In the developing cortex of primates, the SVZ can be further subdivided into the iSVZ and oSVZ based on the robust expansion of basal progenitors.^[^ ^67^, ^68^, ^69^ ^]^ Although the expression of SP0535 increased the population of neural progenitors, there appeared to be no iSVZ‐ or oSVZ‐specific increase. Based on these results, we conclude that the primary reason for cortical enlargement in TG mice is the SP0535‐induced enhancement of progenitor proliferation in the SVZ.

According to previous studies, perturbations of the ATP1A1‐Src complex significantly affects the cell proliferation.^[^ ^39^, ^40^, ^41^, ^42^, ^43^ ^]^ As we identified that SP0535 interacts with ATP1A1, we speculate that such an interaction might affect the ATP1A1‐Src complex. Although we fail to reveal a strong and specific expression correlation of *ATP1A1* and *SP053* in reanalyzing the public scRNA‐seq datasets of various human brain developmental stages, considering that *ATP1A1* is a conserved gene involved in dozens of cellular pathways, such a result is understandable. Overall, although further studies can further clarify the downstream mechanisms of such an interaction, SP0535 in this case can induce phenotypes through affecting ATP1A1 and its regulatory networks.

In this study, we revealed a significant increase in cognitive capability and working memory in SP0535‐TG adult mice. However, there appears to be only some regional aggregation of deep‐layer neurons in the cortex of adult TG mice, which is less potent than the phenotypes of cortical thickening and neuronal increase in TG mice at E15.5. The phenotypes detected at the two stages might need to be reconciled. Recently, studies in nonhuman primates have linked the developing neuronal organization and connectivity to changes in cognitive abilities of the comparable brain structures.^[^ ^70^, ^71^ ^]^ The striking effects of SP0535 in fetal brains of the TG mice can inevitably alter the distribution of neurons and the reconfiguration of neuronal circuits, which further shape cognitive capability and working memory in adult mice. This might be another aspect worthy of investigation in the future.

## 4 Experimental Section

### Animals

Animal housing conditions and all the experimental procedures used in this study were complied with the guidelines of the Institutional Animal Care and Use Committee of the Institute of Zoology, Chinese Academy of Sciences. The animal experimental procedures were in accordance with the Guide for the Care and Use of Laboratory Animals published by the US National Institutes of Health (NIH Publication No. 85‐23, revised 1996). All mice had free access to food and water and were housed in the institutional animal care facility (SPF) with a 12‐h light–dark schedule.

*SP0535*‐HA knockin mice and DPPA3‐Cre TG mice were purchased from the Shanghai Model Organisms Center (Shanghai). Both mouse lines were on the C57BL/6 background. The *SP0535* TG mouse line was constructed using the CRISPR/Cas9 system, and the CAG‐LoxP‐Stop‐LoxP‐*SP0535*‐3x*HA*‐WPRE‐polyA expression cassette was inserted into the Rosa26 locus via homologous recombination. In brief, Cas9 mRNA and gRNA were obtained using in vitro transcription. Then, a homologous recombinant vector (donor vector) was constructed using in‐fusion cloning, which contains a 3.3 kb 5′ homologous arm, a CAG‐LSL‐*SP0535*‐3x*HA*‐WPRE‐polyA cassette, and a 3.3 kb 3′ homologous arm. Finally, the Cas9 mRNA, gRNA, and donor vector were microinjected into fertilized eggs of C57BL/6J mice to obtain F0 mice. The F0 mice were hybridized with DPPA3‐Cre mice to obtain SP0535‐expressing mice. C57BL/6 WT mice were purchased from SPF (Beijing) Biotechnology Corporation.

### Human Cell Culture

The hESC line H9 was maintained in E8 medium (Life Technologies, Carlsbad, USA) on feeder‐free Matrigel (Corning, New York, USA) precoated 6‐well plates or coverslips. The medium was changed every 24 h. The cells were passaged every 6 days and tested for *Mycoplasma* contamination every week.

HEK293T cells were maintained in Dulbecco's modified Eagle's medium (DMEM, Life Technologies) supplemented with 10% fetal bovine serum (Gibco, Carlsbad, USA), 1% nonessential amino acids (Gibco), 1% sodium pyruvate (Gibco), and 0.1% penicillin/streptomycin (Gibco) on gelatin‐coated 6‐well plates or 24‐well plates with coverslips. All cells were maintained at 37 °C in the presence of 5% CO_2_.

### Human Tissue Samples

Human fetal tissue was obtained from the Third Hospital of Peking University, China, following elective pregnancy termination and after obtaining informed written maternal consent with the approval of the University Hospital Ethics Review Committees. The protocols were in compliance with the “Interim Measures for the Administration of Human Genetic Resources” administered by the Ministry of Health of China. A male fetus of 29 gestational weeks (assessed using ultrasound measurement of crown‐rump length) that had not been involved in any other procedure was used in this study. Brain tissue was dissected in ice‐cold artificial cerebrospinal fluid (ACSF) containing NaCl (125 mm), KCl (5 mm), CaCl_2_ (2 mm), MgSO_4_ (1 mm), NaHCO_3_ (25 mm), NaH_2_PO_4_ (1.25 mm), and glucose (20 mm) at pH 7.4, 310 mOsm kg^−1^, in the presence of 95% O_2_ and 5% CO_2_. The right hemisphere was divided coronally and embedded in 4% low‐melting‐temperature agarose in ACSF and cut at 300 µm thickness using a vibratome (Leica Microsystems, Wetzlar, Germany). The VZ, SVZ, and CP cellular zones were carefully microdissected from the brain slices in Hanks' balanced salt solution under a stereoscope (Olympus, Tokyo, Japan) and were subsequently harvested and processed in TRIzol reagent (Invitrogen, Carlsbad, USA) for RNA extraction.

### Isolation of Mouse Brains and Neocortices

For E15.5 fetuses, pregnant mice were anesthetized using isoflurane, and the fetuses were isolated on ice. The brains were dissected in ice‐cold phosphate‐buffered saline (PBS) (Corning) under a stereoscope. For scRNA‐seq, brains were snap‐frozen in liquid nitrogen and then processed for sequencing and analytical procedures. For immunohistochemistry, the brains were directly fixed in 4% paraformaldehyde (PFA) for 24 h, followed by dehydration in 30% sucrose for 48 h. For protein or RNA extraction, the cortices were dissected from fresh brain and washed thrice using PBS or RNase‐free water, followed by ultrasonication in radioimmunoprecipitation assay (RIPA) buffer or TRIzol reagent.

Adult mice were anesthetized using isoflurane and perfused with 0.9% saline for 7–8 min, followed by treatment with 4% PFA for 5 min. For immunohistochemistry, the brains were dissected out and postfixed overnight in 4% PFA at 4 °C, followed by dehydration in 30% sucrose for 48 h.

### Generation of hCOs

Cortical organoids were generated from H9 hESCs using a previously reported protocol^[^ ^49^ ^]^ with some modifications. The hESCs were dissociated to obtain single cells using Accutase for 5 min at 37 °C. In total, 9000 cells were plated on each well of a V‐shaped 96‐well plate (Sumitomo Bakelite) to form a single organoid in medium containing DMEM/F12, 20% KSR, 1% GlutaMAX, 1% nonessential amino acids, *β*‐mercaptoethanol (0.1 mm), the ROCK inhibitor Y27632 (5 µm), the TGF‐*β* inhibitor SB431542 (8 µm), and the BMP inhibitor dorsomorphin (5 µm). The organoids were maintained in 96‐well plates for 5 days and then transferred to ultralow‐attachment 10 cm dishes (Corning) containing neural induction medium supplemented with Neurobasal, 50× B27 supplement, 1% GlutaMAX, 20 ng mL^−1^ EGF, and 20 ng mL^−1^ bFGF. The medium was changed every day. On day 25, the organoids were transferred to neural differentiation medium containing Neurobasal, 50× B27 supplement, 1% GlutaMAX, 20 ng mL^−1^ BDNF, 20 ng mL^−1^ NT3, 10 ng mL^−1^ GDNF, and 10 ng mL^−1^ IGF1. The medium was changed every 3 days.

### Generation of SP0535 KO Cell Lines

The CRISPR/Cas9‐based KO cell line was established following a reported method.^[^ ^72^ ^]^ Briefly, to establish the gRNA expression vector, a pair of sgRNAs capable of deleting a 200 bp‐long DNA sequence from *SP0535* exon 3 was designed using the CRISPER design tool (http://crispr.mit.edu) to generate frameshift mutations. The sgRNAs were annealed and ligated into the pMini‐sgRNA vector. For nucleofection (Lonza, Basel, Switzerland), the hESCs were cultured to 80% confluence, and Y27632 (10 µm) was added to the medium 1 h before nucleofection. Next, pMini‐sgRNA plasmid (2.5 µg)^[^ ^72^ ^]^ and pMax‐Cas9 plasmid (5 µg) (Lonza) were transfected into 2 × 10^6^ suspended hESCs. After nucleofection, the hESCs were plated on Matrigel‐precoated 6‐well plates in E8 medium containing Y27632 (10 µm). After 48 h, the cells were dissociated with Accutase, further dispersed into single cells, and then washed three times with PBS. Subsequently, the cells were sorted using flow cytometry (BD Biosciences, Franklin Lakes, USA), collected in E8 medium supplemented with Y27632 (10 µm), and then seeded on Matrigel‐precoated 6‐well plates. After 7–10 days, single colonies were picked for PCR genotyping. Two *SP0535* KO H9 cell lines were used in this study.

For each sgRNA, the top five putative off‐target sites were selected according to the MIT CRISPR design tool (http://crispr.mit.edu). Then, PCR primers were designed based on the sequences of the flanking regions of these sites, and 400–600 bp DNA fragments with the target regions were amplified (Table S4, Supporting Information), purified, and sequenced to investigate whether off‐target effects existed at these regions.

### Cell Transfection

After reaching 80% confluence, HEK293T cells were transfected with the indicated plasmids (3 µg per transfection for 6‐well plates and 1 µg per transfection for 24‐well plates) (Table S5, Supporting Information) using GenEscort (Wisegen, Nanjing, China) according to the manufacture's protocol. To harvest *SP0535*‐expressing/control HEK293T cells, 0.25% trypsin‐EDTA was used for 3 min to dissociate cells, followed by centrifugation at 1100 rpm for 3 min.

### EdU‐Click‐iT Assay

To label hESCs, EdU (10 µm, Life Technologies) was added to the E8 medium. After 30 min, the samples were fixed using 4% PFA and used for the Click‐iT assay using the Click‐iT Plus EdU imaging kit (Thermo Fisher Scientific, Carlsbad, USA) in the dark following the manufacturer's instructions. For E15.5 fetuses, EdU (50 mg kg^−1^ body weight, 1:1 mixed with 0.9% saline) was injected intraperitoneally into pregnant mice 2 h before sacrifice. All EdU‐labeled samples were directly used for immunofluorescence staining.

### Identification of Human‐Specific De Novo Genes

A list of 45 human‐specific de novo genes was identified based on the synteny‐based approach as previously reported.^[^ ^27^ ^]^ To further clarify the contributions of these new genes to human uniqueness in brain development, first genes with relatively higher expression in brain tissues and/or those with differential expression in fetal brain development were focused (Figure S1B, Supporting Information). Also genes with a coding region shorter than 100 amino acids were removed. Moreover, the selection signatures of these new genes were also considered to prioritize them with adaptive functions. According to these criteria, *ENSG00000203930* (*SP0535*) was selected for in‐depth functional study in cortical organoids and TG mice in this study.

### scRNA‐seq and Analysis

E15.5 brains were removed on ice and dissociated using papain diluted in RNase‐free water for 20 min at 37 °C. The cell suspension was filtered using a 40 µm cell strainer and centrifuged at 200 × *g* for 5 min. The cells (100 000 cells mL^−1^) were washed thrice with ice‐cold RNase‐free water (10 mL) at 200 × *g* for 5 min and then resuspended in ice‐cold RNase‐free water containing 1% bovine serum albumin (BSA, Sigma, St. Louis, USA); 10× Genomics chips were loaded to recover 7000–10 000 cells. cDNA amplification, library construction, and raw data processing and analysis were performed following the 10× Genomics protocols.^[^ ^35^ ^]^

For quality control of the single‐cell data, the raw gene expression matrixes generated from each sample were aggregated using CellRanger (v5.0.1). Downstream analysis was performed using the R package Seurat (v3.2.0). Quality control was applied to cells based on the number of detected genes and the proportion of mitochondrial reads per cell. In particular, cells with less than 200 detected genes or cells with >90% of the proportion of maximum genes were filtered out. For the mitochondrial metric, the cells were sorted in descending order of mitochondrial read ratio, and the top 15% of cells were filtered out. Potential doublets were identified and removed using DoubletDetection. Cell cycle analysis was performed using the CellCycleScoring function in the Seurat program. The gene expression dataset was normalized, and principal component analysis (*n* = 15) was performed using only the 2000 highly variable genes in the dataset.

UMAP was then used for 2D visualization of the resulting clusters. For each cluster, the marker genes were identified using the FindAllMarkers function as implemented in the Seurat package (logfc. threshold > 0.25, minPct > 0.1, and Padj ≤ 0.05). Then, the clusters were remarked to a known cell type using the SCSA (https://github.com/bioinfo‐ibms‐pumc/SCSA) method. Differentially expressed genes across different samples were identified using the FindMarkers function in Seurat with the parameters “logfc. threshold > 0.25, minPct > 0.1, and Padj ≤ 0.05.”

Functional enrichment analyses were performed using phyper on the basis of GO terms and KEGG pathways (V93.0).

### Bulk Tissue Gene Expression from the GTEx Database

GTEx Release V8 (dbGaP Accession phs000424.v8. p2) was used to retrieve the tissue expression profiles of genes, in which the expression levels were shown in transcripts per million.

### Immunofluorescence Staining and Confocal Imaging

For mouse brain sections, the dehydrated brains were placed in tissue base molds and embedded in OCT compound at −20 °C. Then, cryosectioning was performed to obtain 25‐µm slices using a freezing microtome. The slices were washed three times with PBS (5 min each time) at 25 °C and incubated in blocking buffer (PBS, 5% BSA, 1% Triton X‐100) for 1 h. Then, the samples were incubated overnight at 4 °C with primary antibodies diluted in 1% BSA and 1% Triton X‐100 in PBS. The next day, the samples were washed three times in PBS for 5 min each and subsequently incubated with secondary antibodies at room temperature for 90 min, diluted in 1% BSA and 1% Triton X‐100 in PBS. After washing three times with PBS, the nuclei were counterstained with Hoechst (Invitrogen) at room temperature for 10 min and mounted on glass slides.

For hCO sections, the organoid samples were collected in 2 mL Eppendorf tubes and fixed overnight using 4% PFA at 4 °C. The subsequent procedure was the same as described above for brain sectioning and immunofluorescence staining.

For coverslips, hESC or HEK293T cultures were fixed using 4% PFA at room temperature for 30 min and washed three times with PBS (5 min for each wash). The subsequent procedure was the same as that described for brain tissue immunofluorescence staining. Images were captured using a Carl Zeiss LSM880 confocal microscope and processed using ZEN 2012 software. The cells were counted using Imaris software. Cortical thickness and organoid area were measured using ImageJ software. At least three images were captured for every experimental condition. The details regarding the antibodies used and their catalog numbers and dilutions are presented in Table S2, Supporting Information.

### Western Blotting

Samples were lysed using RIPA buffer containing a protease inhibitor (Invitrogen). Protein was quantified using the bicinchoninic acid (BCA) assay (Invitrogen), and each lysate (25 µg) was loaded per lane of a NuPAGE 4–12% bis–tris protein gel (Thermo Fisher Scientific). Samples were separated at 120 V for 40 min and then transferred onto polyvinylidene fluoride (PVDF) membranes. The membranes were blocked using 4% BSA in tris‐based saline with Tween 20 (0.05% TBST) for 1 h, followed by overnight incubation with primary antibodies at 4 °C. The next day, the membranes were washed three times with 0.05% TBST (5 min each) and then incubated with horseradish peroxidase‐conjugated secondary antibodies for 90 min at room temperature. The protein bands were visualized using the SuperSignal West Pico PLUS chemiluminescent substrate (Thermo Fisher Scientific), and the blot image was captured using the Tanon 5200 Automatic chemiluminescence image analysis system. Band intensities were quantified using ImageJ for three independent experiments.

### Immunoprecipitation and Coimmunoprecipitation

Cerebral hemispheres of E15.5 mice or transfected HEK293T cells were lysed in IP RIPA buffer (Invitrogen) containing a protease and phosphatase inhibitor cocktail (Invitrogen). Protein was quantified using the BCA assay, and each protein lysate (1 mg) was incubated with HA or FLAG antibodies (1 µg) with gentle rotation for 1 h, following which A/G agarose beads (20 µL; Santa Cruz Biotechnology, Santa Cruz, USA) were added to the lysate and incubated overnight at 4 °C with gentle rotation. The next day, the beads were collected via centrifugation at 2500 rpm for 5 min. After three washes (15 min each time with gentle rotation at 4 °C), the beads were resuspended in IP RIPA buffer and loading buffer, boiled, and centrifuged to obtain the protein precipitate with specific antibodies. For immunoprecipitation, the samples were separated using sodium dodecyl sulfate‒polyacrylamide gel electrophoresis, followed by mass spectrometry. For coimmunoprecipitation, the proteins were transferred to a PVDF membrane. The subsequent steps were the same as those described above for Western blotting.

### RNA Extraction, Reverse Transcription, and Quantitative‐PCR

Cells and tissues were homogenized, and total RNA was extracted using TRIzol. Then, first‐strand cDNA was generated using the PrimeScript RT reagent kit with gDNA digest (Yeasen, Shanghai, China) and random hexamers, following the manufacturer's instructions. RT‒qPCR was performed using the Power SYBR Green PCR master mix (Yeasen) on the ABI QuantStudio 6 Flex real‐time PCR system (Applied Biosystems, Carlsbad, USA). The primers used for RT‒qPCR are listed in Table S3, Supporting Information.

### Behavioral Tests

WT and heterozygous *SP0535*‐TG male mice were tested at 10–12 weeks of age. Before every test, the mice were handled for 3 days to allow them to adapt to the experimental environment. The testing arenas, used objects, and animal enclosures were cleaned with 75% ethanol between each tested animal to remove olfactory traces. The daily experimental time interval was controlled at 24 h. All data were calculated and analyzed using EthoVision (Noldus, Wageningen, Netherlands) software.

### Open Field

Mice were gently placed in a square arena (40 cm wide × 40 cm long × 40 cm high) comprising an inside square (200 mm × 200 mm) as the “center area” and an outside square as the “surrounding area” and allowed to explore freely for 5 min to test their spontaneous exploration ability. The distance traveled and the movement tracks in the center and surrounding areas were measured using the EthoVision tracking system.

### Novel Object Recognition

The tests were performed according to a reported method.^[^ ^73^ ^]^ Briefly, mice were allowed to explore an open‐field box with two identical objects (cylinders) placed opposite to the starting point for 5 min. The next day, the mice were allowed to explore one familiar object (cylinder) and one novel object (cuboid) for 5 min. The duration of mice touching each object was recorded using the EthoVision tracking system. The discrimination ratio is indicated in the related figure legends.

### Morris Water Maze

Tests were conducted in a circular pool 150 cm in diameter filled with water (23 ± 2 °C) to a depth of 22 cm. A circular platform (8 cm in diameter) was placed 2 cm beneath the water level. The water maze was divided into four logical quadrants (north, south, east, and west), which acted as the starting positions for the mice. All animals underwent the learning, probe test, reversal learning, and probe test sessions (shown in Figure 5f). Latency was calculated from the average of the four quadrants. In the reversal learning trials and probe tests, the platform was placed at a position that was opposite to its location in the normal learning trials. The latency to find the platform during the training and test trials was recorded using the EthoVision tracking system.

### Eight‐Arm Radial Maze

The procedures were performed as described previously.^[^ ^74^ ^]^ Prior to the experiment (day −1), the mice were fasted for 24 h. Then, they were provided milk chocolate pellets weighing 0.2 g as a food reward. On day 0, the food pellets were placed in all eight arms (three pellets in each arm), and mice were allowed to freely explore for 10 min. This was repeated once after 1 h. On days 1–5, food rewards (three pellets in each arm) were placed only in arms 1, 3, 4, and 7 (Figure 5j). The latency to finish all food pellets (task fulfillment) was recorded to reflect working memory.

### Three‐Chamber Social Interaction Test

The test was performed as described previously.^[^ ^56^ ^]^ Individual test mice were allowed to habituate to the environment with two empty wire cages for 10 min. After habituation, an age‐matched unfamiliar male mouse was placed in a random wire cage. The test mouse was placed in the middle chamber and allowed to explore the chamber for 5 min. Then, a second unfamiliar mouse was placed in another wire cage and allowed to explore the chamber for 5 min.

### RotaRod Test

The tests were performed according to a reported protocol.^[^ ^55^ ^]^ On day 1, the mice were placed on the rotating bar of the autoaccelerating rotating rod fatigue apparatus and allowed to acclimatize to 5 to 40 rpm in 5 min, followed by 40 rpm for 10 min, which was repeated after every 2‐h interval. On days 2–3, the mice were trained at the same 40 rpm speed for 10 min, and the procedure was repeated after an interval of 2 h. Tests were performed on day 4 to record the latency of the first fall of the mice from the bar within 5 min.

### Electrophysiology

Brains from WT and TG mouse littermates (8–12 weeks old) were rapidly removed and placed in an ice‐cold NMDG‐based solution consisting of NMDG (92 mm), KCl (5 mm), NaH_2_PO_4_ (1.3 mm), NaHCO_3_ (26 mm), CaCl_2_ (0.5 mm), MgCl_2_ (10 mm), thiourea (2 mm), Na‐ascorbate (5 mm), and D‐glucose (25 mm) (pH adjusted to 7.4 with HCl). Brain slices (400‐µm thick) were obtained using a vibratome slicer (Campden Instruments, 7000 smz). The brain slices were recovered in ACSF for at least 30 min at 30 °C prior to electrophysiological recordings. The ACSF solution contained the following: NaCl (125 mm), KCl (3.25 mM), MgCl_2_ (1.5 mm), NaH_2_PO_4_, 25 NaHCO_3_ (1.25 mm), glucose (10 mm), and CaCl_2_ (2.5 mm). All experiments were performed at room temperature.

fEPSPs were recorded using a multiclamp 700B amplifier (Molecular Devices, Sunnyvale, USA) and an Axon Digidata 1440A digitizer (Molecular Devices) in the cortical L2 + 3 region with stimulation electrodes positioned within the cortical L4 region, and were induced at 0.033 Hz. The slices were maintained in ACSF, which was equilibrated with 95% O_2_ and 5% CO_2_ (pH 7.4). A glass electrode (3–4 MΩ, Sutter Instrument Co., Novato, USA) filled with ACSF was used for recording the fEPSP signals. After at least 15 min of stable baseline recording, LTP was induced by HFS (100 Hz, 1 s). The data were sampled at 10 kHz and filtered at 2 kHz. The fEPSP traces were obtained and analyzed using pClamp 10.6 and Clampfit 10.6 (Axon Instruments, Sunnyvale, USA), respectively.

### Cell Cycle Assay

At day 0, 80 000 HEK293T cells were seeded on a 6‐well plate. Three replicates of each well were performed in the control and overexpression groups, and cell counts were obtained daily thereafter. After 7 days, the growth curve was plotted and fitted as an exponential function, and the time required for doubling of the cell number in a cell cycle was calculated.

### Statistical Analysis

Data were presented as the mean ± SEM or mean ± SD (as indicated in the figure legends). Statistical analyses were performed using Prism 8 software (GraphPad). An unpaired two‐tailed Student's *t*‐test was used to calculate the significance of differences between two groups. The numbers of replicates and the *p* value of each statistical test are indicated in the figure legends and Table S7, Supporting Information.

### Ethics Approval Statement

The animal housing conditions and all the experimental procedures used in this study complied with the guidelines of the Institutional Animal Care and Use Committee of the Institute of Zoology, Chinese Academy of Sciences (IOZ‐IACUC‐2021‐105). The animal experimental procedures were in accordance with the Guide for the Care and Use of Laboratory Animals published by the US National Institutes of Health (NIH Publication No. 85‐23, revised 1996).

Human fetal tissue was obtained from the Third Hospital of Peking University, China, following elective pregnancy termination with the approval of the University Hospital Ethics Review Committees. Biomedical ethics was approved by the Institute of Zoology, Chinese Academy of Sciences (DSYL‐2022‐007). The protocols were in compliance with the “Interim Measures for the Administration of Human Genetic Resources” administered by the Ministry of Health of China.

### Patient Consent Statement

Human fetal tissue was obtained from the Third Hospital of Peking University, China, following elective pregnancy termination and after obtaining informed written maternal consent.

## Conflict of Interest

The authors declare no conflict of interest.

## Supporting information

Supporting Information

Supporting Information

## Data Availability

The data that support the findings of this study are available from the corresponding author upon reasonable request.
