# Supplementary material for: A Human‐Specific De Novo Gene Promotes Cortical Expansion and Folding
Source: Adv Sci (Weinh). 2023 Jan 13;10(7):2204140. doi: 10.1002/advs.202204140 (PMC9982566; doi:10.1002/advs.202204140)
Supplement: Supplementary file 1 — Supporting Information [file ADVS-10-2204140-s002.pdf]

ADVANCED SCIENCE
Open Access

Supporting Information

for *Adv. Sci.*, DOI 10.1002/advs.202204140

# A Human-Specific De Novo Gene Promotes Cortical Expansion and Folding

*Jianhuan Qi, Fan Mo, Ni A. An, Tingwei Mi, Jiaxin Wang, Jun-Tian Qi, Xiangshang Li, Boya Zhang, Longkuo Xia, Yingfei Lu, Gaoying Sun, Xinyue Wang, Chuan-Yun Li* and Baoyang Hu**

# Supporting Information

## A Human-specific De Novo Gene Promotes Cortical Expansion and Folding

*Jianhuan Qi†, Fan Mo†, Ni A. An†, Tingwei Mi, Jiaxin Wang, Jun-Tian Qi, Xiangshang Li, Boya Zhang, Longkuo Xia, Yingfei Lu, Gaoying Sun, Xinyue Wang, Chuan-Yun Li*, and Baoyang Hu**

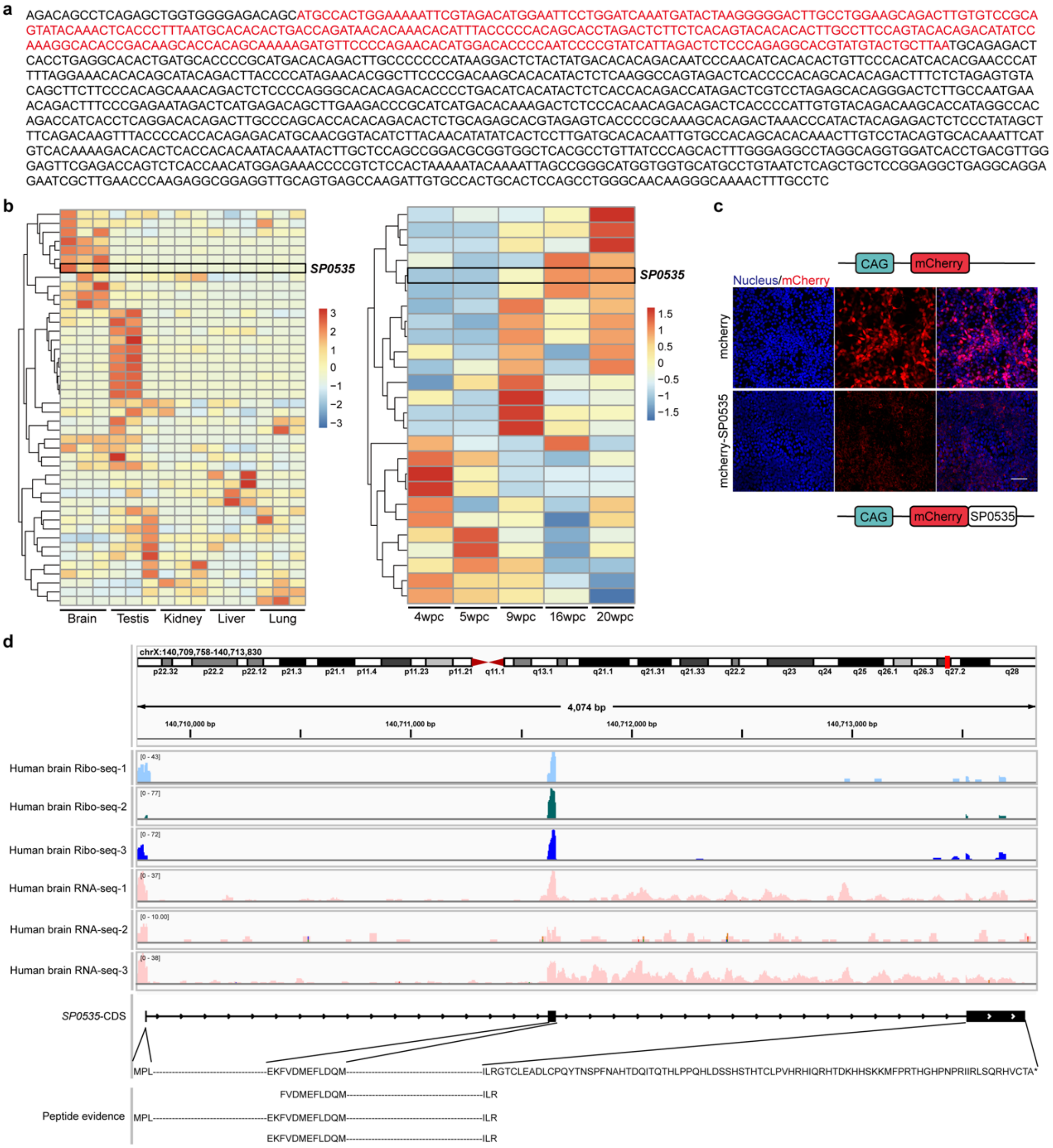

**Figure S1.** Additional information of de novo SP0535 in locus of *ENST00000370535*, related to Figure 1. (a) DNA sequence of mature transcript of *ENST00000370535* and the CDS sequence of *SP0535* (red). (b) The expression profiles of 45 human de novo genes. The expression profiles of de novo genes across five different human tissues (left; brain, kidney, liver, lung, and testis) and different developmental stages during brain development (right). 4wpc-20wpc: postconceptional weeks 4-20 in fetal brain development. The black boxes highlight the expression profiles of *ENSG00000203930.* Genes not expressed in these samples were not included. (c) Images of HEK293T cells transfected with mcherry or mcherry-SP0535. Schematic diagram of the vector construction is shown on side. Scale bar, 50μm. (d) Translational evidence of *SP0535* in human. The CDS structure of *SP0535* was shown, with the items of evidence supporting the translational expression of this new gene aligned accordingly, including the reads coverage of Ribo-seq data of the public human brain (Human brain Ribo-seq-1, Human brain Ribo-seq-2, Human brain Ribo-seq-3), the peptide identified by large-scale mass spectrometry (Peptide evidence, retrieved from PRIDE, PeptideAtlas, ProteomicsDB and Human Proteome Map database).

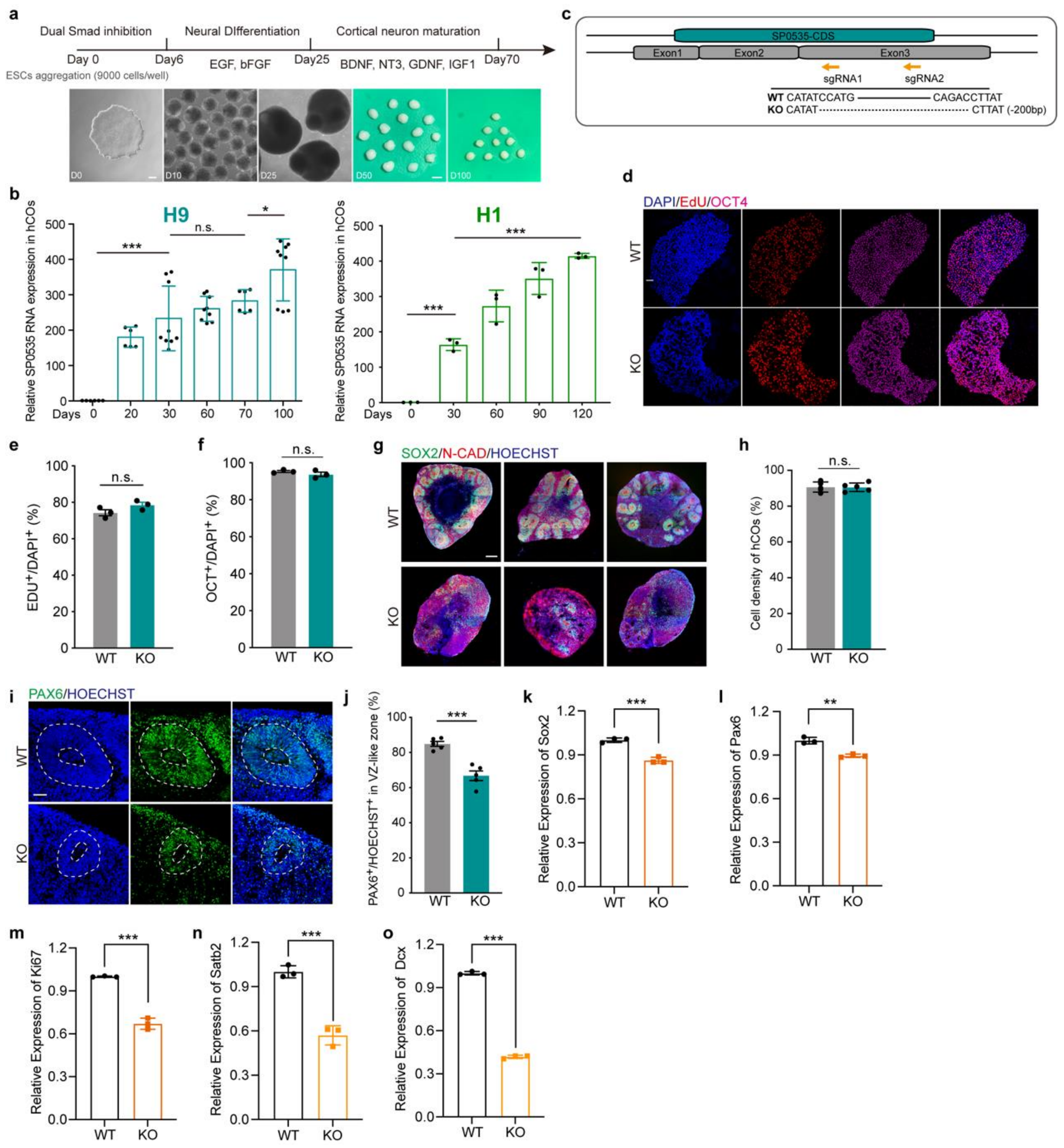


**Figure S2.** Cortical development is impaired in *SP0535* knockout hCOs, related to Figure 2. (a) Schematic procedure for hESC-derived cortical organoid differentiation. Scale bar, 200µm (Left) and 2mm (Right). (b) Relative expression of *SP0535* in different days of H9/H1 human embryonic stem cell derived cortical organoid. Data are presented as mean ± SD ($^{*}p < 0.05$ and $^{***}p < 0.001$, Student's *t* test). (c) Schematic of CRISPER-Cas9 based *SP0535* knockout hESC lines. (d) Immunostaining for EdU and OCT4 in wild-type and *SP0535* knockout ESCs. Scale bar, 100µm. (e and f) Quantification for the percentage of EdU$^{+}$(e) and OCT4$^{+}$(f) cells in wild-type and *SP0535* knockout ESCs (n=3 each group). Data are presented as mean ± SEM (unpaired two-tailed Student's *t* test). (g) Immunostaining for SOX2 and

N-cadherin in 5-week wild-type and *SP0535* knockout hCOs. Scale bar, 300μm. (h) Cell density of wild-type and KO hCOs (n=5 each group). Data are presented as mean ± SEM (unpaired two-tailed Student's *t* test). (i) Immunostaining for PAX6 in 5-week wild-type and SP0535 knockout hCO VZ-like zones. Scale bar, 100μm. (j) Quantification for the percentage of $PAX6^+$ in 5-week wild-type and *SP0535* knockout hCOs (n=5 each group). Data are presented as mean ± SEM ($^{***}p < 0.001$, Student's t test). (k-m) Relative expression of *SOX2* (j), *PAX6* (k), and *KI67* (l) in 5-week wild-type and *SP0535* knockout hCOs (n=3 each group). Data are presented as mean ± SD ($^{**}p < 0.01$ and $^{***}p < 0.001$, Student's *t* test). (n and o) Relative expression of *SATB2* (m) and *DCX* (n) in 10-week wild-type and *SP0535* knockout hCOs (n=3 each group). Data are presented as mean ± SD ($^{***}p < 0.001$, unpaired two-tailed Student's *t* test).

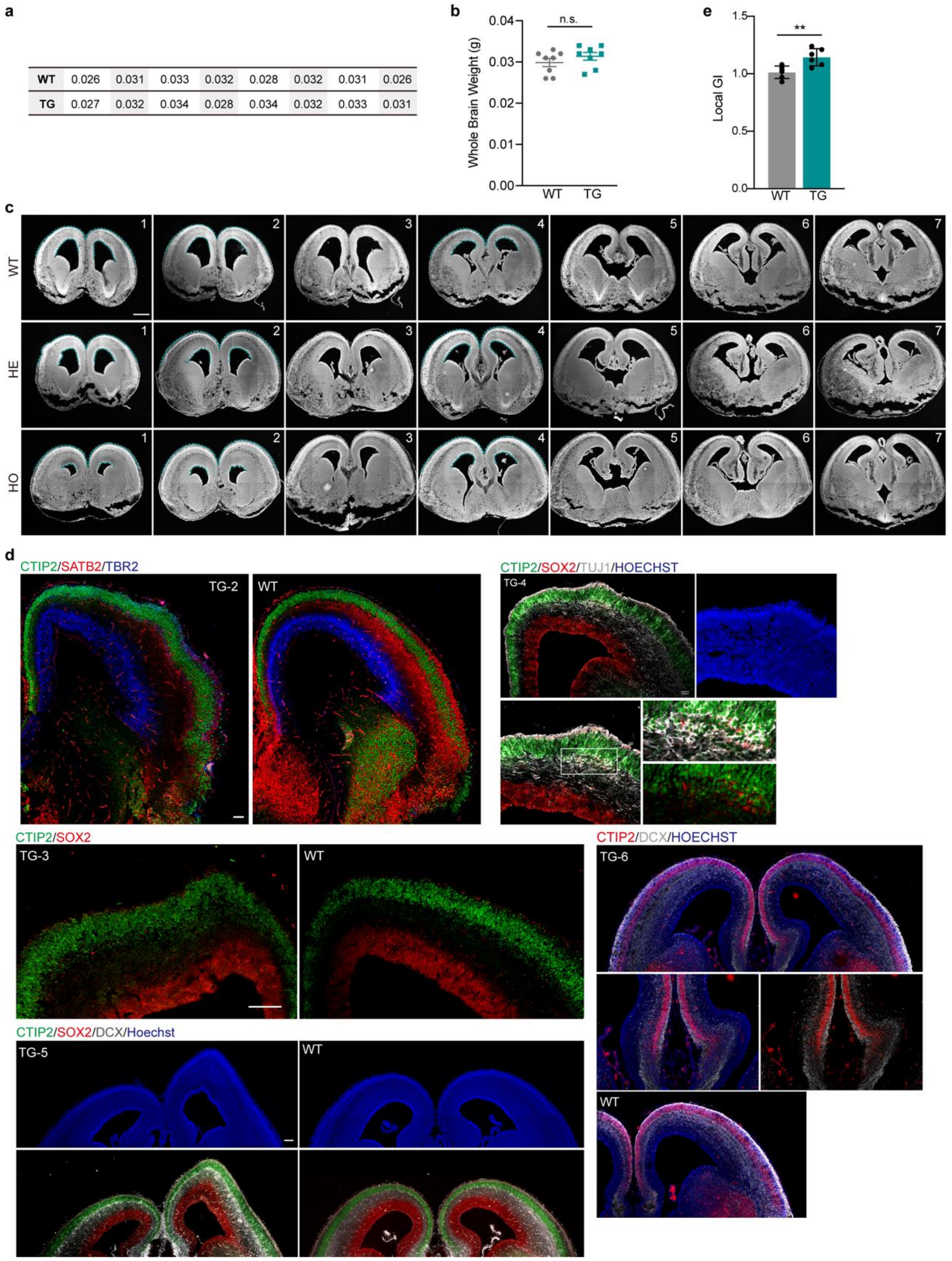


| | | | | | | | | |
|---|---|---|---|---|---|---|---|---|
| **WT** | 0.026 | 0.031 | 0.033 | 0.032 | 0.028 | 0.032 | 0.031 | 0.026 |
| **TG** | 0.027 | 0.032 | 0.034 | 0.028 | 0.034 | 0.032 | 0.033 | 0.031 |

**Figure S3.** Cortex expansion and sulci& gyri formation in E15.5 TG mice, related to Figure 3. (a and b) whole brain weight of E15.5 wild-type and TG mice (a) and quantification (b). (c) Cortical thickness in seven positions of wild-type and TG mice, including homozygotes and heterozygotes. Scale bar, 500μm. (d) Some other cases of sulci& gyri structure in E15.5 *SP0535* knockin mice. Scale bar, 100μm. (e) Local GI

of E15.5 wild-type and TG mice. Data are presented as mean ± SEM. ($^{**}p < 0.01$, unpaired two-tailed Student's *t* test).

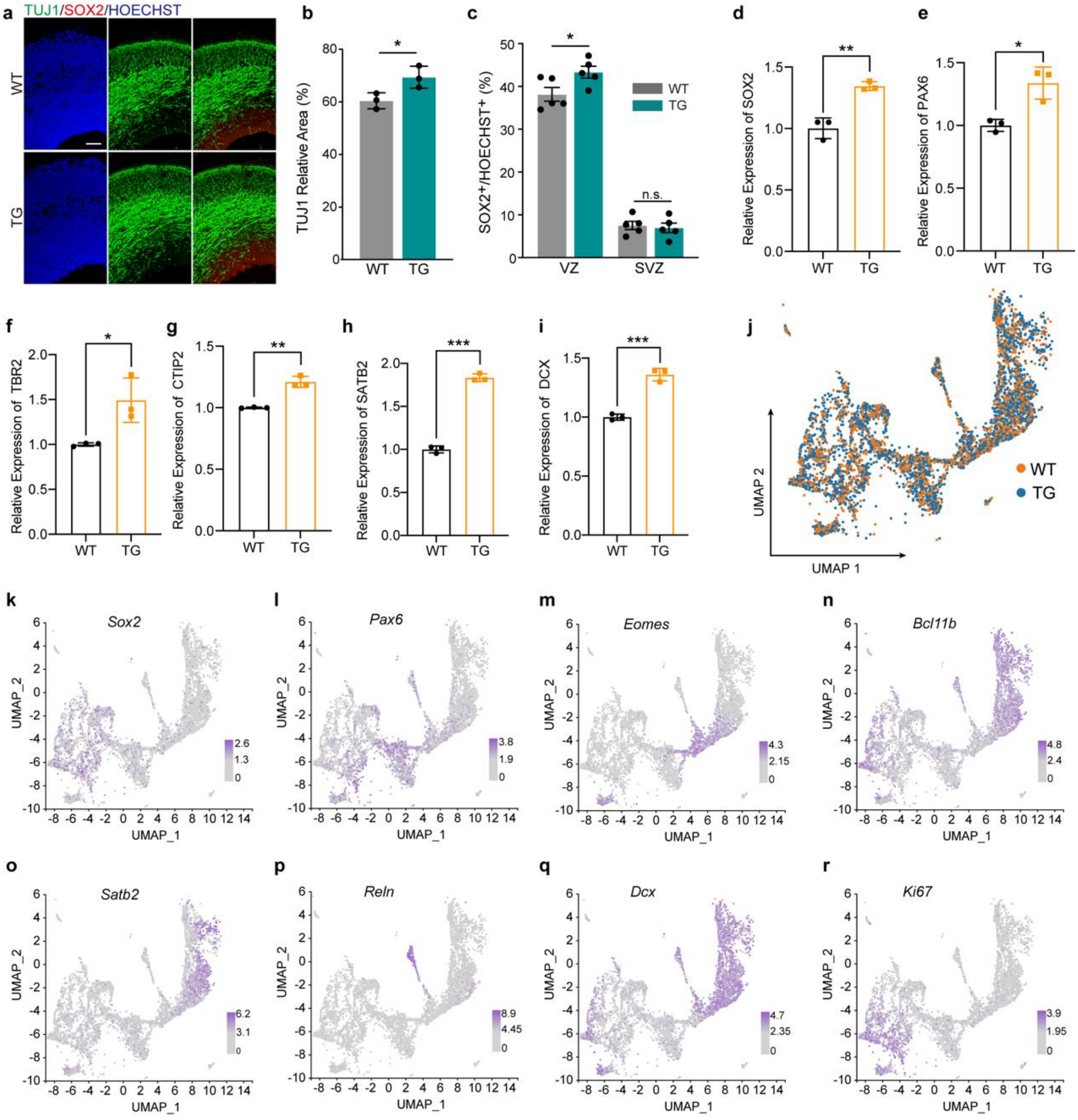


**Figure S4.** *SP0535* knockin induces neural progenitor markers expression, related to Figure 4. (a) Immunostaining for TUJ1 in E15.5 wild-type and TG mice. Scale bar, 100μm. (b) Quantification for the relative area of $TUJ1^{+}$ cells in E15.5 cortices (n=3 each group). Data are presented as mean ± SEM ($^{*}p < 0.05$, unpaired two-tailed Student's *t* test). (c) Quantification of $SOX2^{+}$ cell in VZ and SVZ, respectively. Data are presented as mean ± SEM ($^{*}p < 0.05$, unpaired two-tailed Student's *t* test). (d-i) Relative expression of *Sox2* (d), *Pax6* (e), *Tbr2* (f), *Ctip2* (g), *Satb2* (h), and *Dcx* (i) in E15.5 wild-type and TG mice. Data are presented as mean ± SD ($^{*}p < 0.05$, $^{**}p < 0.01$,

and ${}^{***}p < 0.001$, unpaired two-tailed Student's *t* test). (j) UMAP visualization of scRNA-seq from E15.5 WT and TG cortices phenotype.

(k-r) Representative markers in E15.5 wild-type and TG mouse cortices sc-RNAseq clustering, including *Sox2* (k), *Pax6* (l), *Tbr2* (m), *Ctip2* (n), *Satb2* (o), *Reln* (p), *Dcx* (q), and *Ki67* (r).

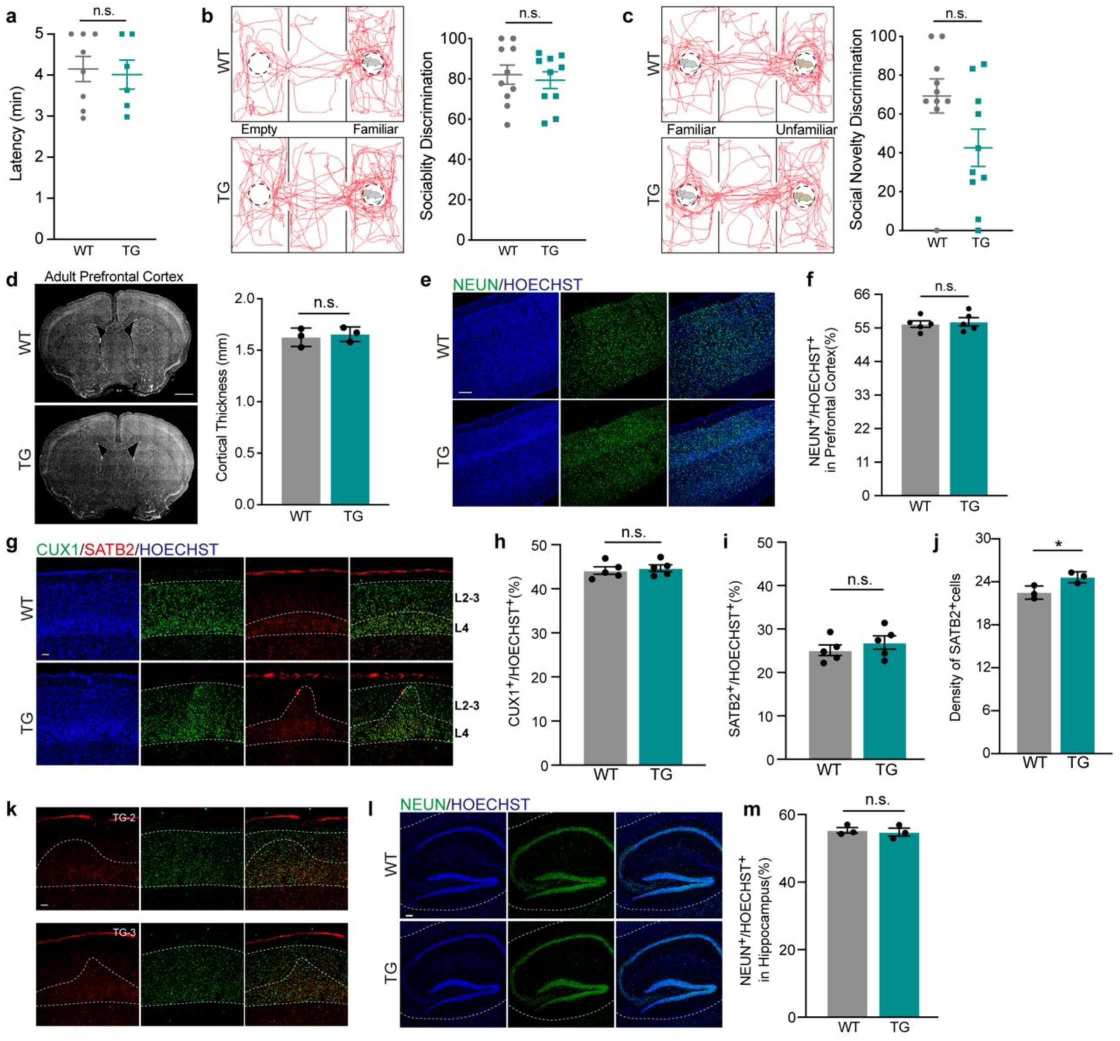


**Figure S5**. *SP0535* knockin has no effect on other performance in mice, related to Figure 5. (a) Latency of wild-type and *SP0535* knockin mice in Rota-rod test ($n^{WT}=8$ and $n^{TG}=6$). (b) Sociability discrimination of wild-type and TG mice in Three-chamber test after twice habituation (n=10 each group). Sociability discrimination is calculated by dividing the time approaching the cage with mouse by the time approaching two cages. Representative moving paths are shown on the left.

(c) Social novelty discrimination of wild-type and TG mice (n=10 each group). Social novelty discrimination is calculated by dividing the time approaching the cage with unfamiliar mouse by the time approaching two cages. Representative moving paths are shown on the left. (d) Representative images and quantifications of adult mouse cortex. (e) Immunostaining for NEUN in wild-type and TG adult prefrontal cortices. Scale bar, 100μm. (f) Quantification for the percentage of $NEUN^+$ cells in adult prefrontal cortices (n=5 each group). (g) Immunostaining for CUX1 and SATB2 in wild-type and SP0535 knock-in adults. Scale bar, 100μm. (h and i) Quantification for the percentage of $CUX1^+$ (g) and $SATB2^+$(h) cells in adult prefrontal cortices (n=5 each group). (j) Density of $SATB2^+$ cells aggregated regions (n=3 each group). (k) Other cases of the aggregation of deep layer neurons. Scale bar, 100μm. (l) Immunostaining for NEUN in wild-type and TG adult hippocampi. Scale bar, 100μm. (m) Quantification for the percentage of $NEUN^+$ cells in adult hippocampi (n=3 each group). Data are presented as mean ± SEM (unpaired two-tailed Student's *t* test).

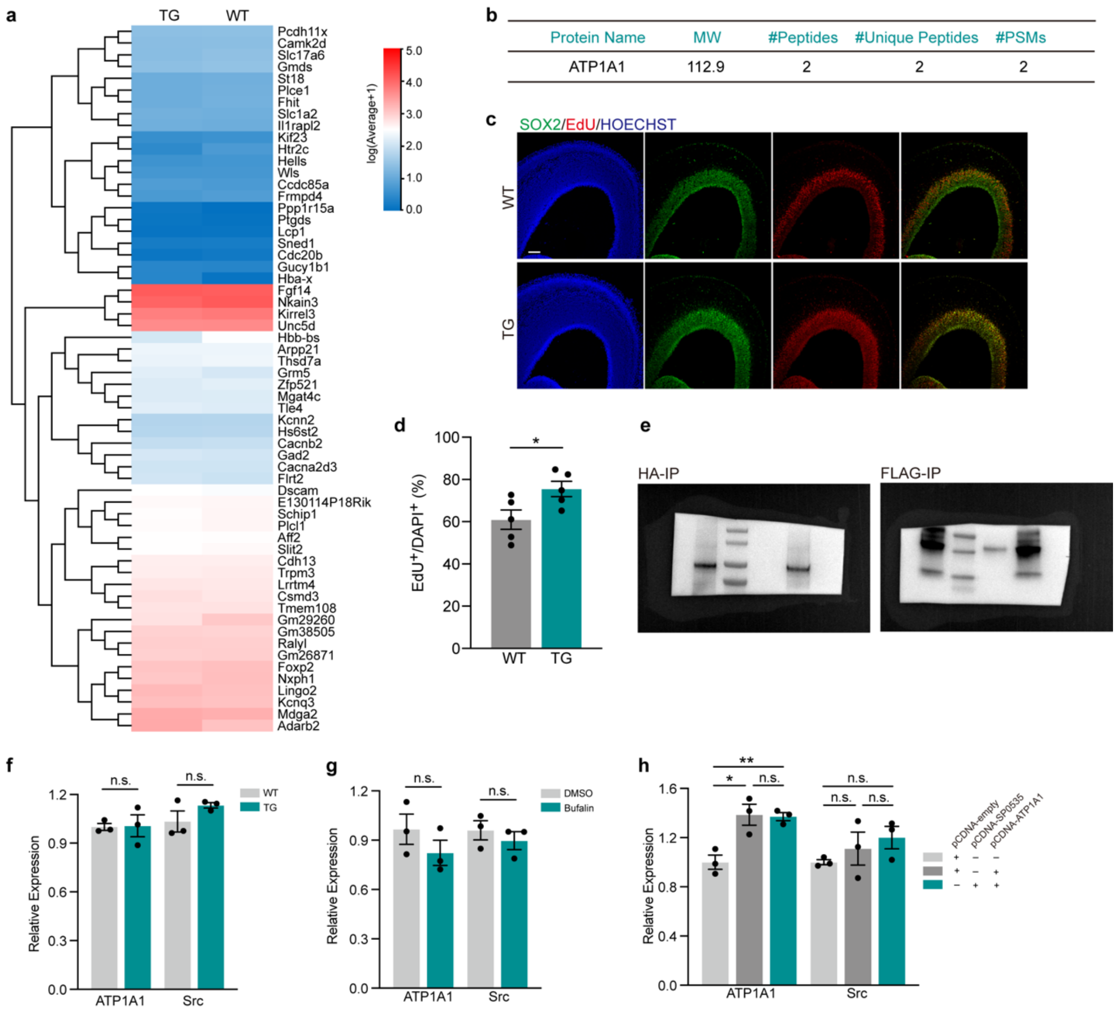


**Figure S6.** SP0535 regulates cell proliferation in E15.5 cortices, related to Figure 6.

(a) Differential gene heatmap of RGCs in sc-RNAseq. |log2FC|>=0.5, adjusted P-value<=0.05. (b) MS analysis result of ATP1A1. (c) Co-immunostaining for EdU and SOX2 in E15.5 wild-type and SP0535 knockin mice. Scale bar, 100µm. (d) Quantification for the percentage of $EdU^+$ cells in $SOX2^+$ cells in E15.5 cortices (n=5 each group). (e) Full blot images of Figure 6F. (f) Western blot quantifications of ATP1A1 and Src, related to Figure 7d (n=3 each group). (g) Western blot quantifications of ATP1A1 and Src, related to Figure 7e (n=3 each group). (h) Western blot quantifications of ATP1A1 and Src, related to Figure 7f (n=3 each group). Data are presented as mean ± SEM ($^{*}p < 0.05$, and $^{**}p < 0.01$, unpaired two-tailed Student's *t* test).

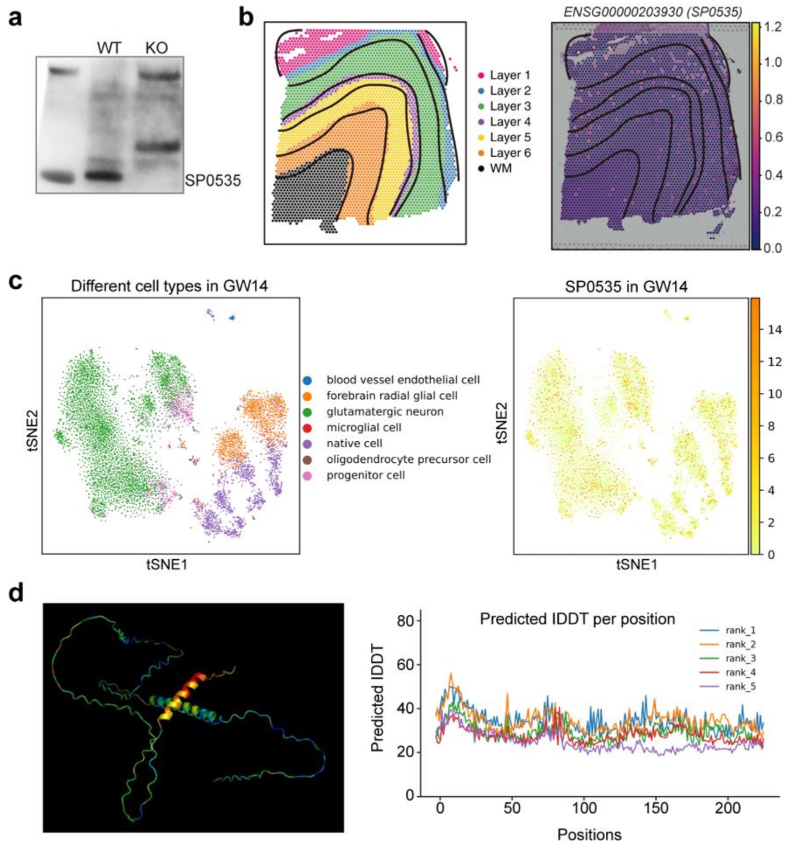


**Figure S7**. Expression of SP0535. (a) Western blotting of D35 wild-type and KO hCOs against SP0535 using generated antibody. (b) Spatial expression of SP0535 in prefrontal cortex of adult human brain PFC. Left: supervised annotation of PFC layers. Right: spatial expression of *SP0535* in the labeled layers. (c) The expression profile of *SP0535* in human GW14 sample. (d) The 3D structure prediction of SP0535 by Alphafold2. Left, the prediction model of SP0535. Right, the predicted IDDT (A local superposition-free score for comparing protein structures and models using distance difference tests) value of SP0535 model.

**Table S1 Summary of studies reporting SP0535 as a human-specific de novo gene.**

| Original studies reporting de novo genes | Translation evidence |
|---|---|
| Xie *et al*., 2012 | MS |
| Chen *et al*., 2015 | MS |
| Shao *et al*., 2019 | MS |
| Dowling *et al*., 2020 | MS & Ribo |
| An *et al*., 2022 | MS & Ribo |

MS, Mass Spectrometry; Ribo, Ribo-seq.

**Table S2 Antibodies for Immunofluorescence Staining**

| Antibodies | Source | Identifier | Dilution |
|---|---|---|---|
| Anti-SOX2 | Goat | AF2018 | 1:500 |
| Anti-PAX6 | Rabbit | 901301 | 1:500 |
| Anti-PAX6 | Mouse | sc32766 | 1:100 |
| Anti-EOMES | Rabbit | ab23345 | 1:200 |
| Anti-BCL11B | Rat | ab18465 | 1:800 |
| Anti-BCL11B | Rabbit | PA5-83952 | 1:500 |
| Anti-SATB2 | Mouse | ab51502 | 1:100 |
| Anti-N-CADHERIN | Rabbit | ab18203 | 1:500 |
| Anti-KI67 | Rabbit | ab9260 | 1:500 |
| Anti-NESTIN (mouse) | Mouse | MAB535 | 1:500 |
| Anti-NESTIN (human) | Mouse | MAB5326 | 1:500 |
| Anti-ATP1A1 | Mouse | ab7671 | 1:500 |
| Anti-HA | Rabbit | ab9110 | 1:1000 |
| Anti-FLAG | Mouse | F1804 | 1:1000 |
| Anti-mcherry | Rabbit | ab167453 | 1:1000 |
| Anti-PH3 | Rabbit | 9701 | 1:500 |
| Anti-PH3 | Rat | ab10543 | 1:800 |

| | | | |
|---|---|---|---|
| Anti-NEUN | Rabbit | Ab177487 | 1:500 |
| Anti-CUX1 | Rabbit | 11733-1-AP | 1:200 |
| Anti-OCT4 | Mouse | 611203 | 1:1000 |

**Table S3 Primers for genotyping and RT-qPCR**

| Target | Sequence |
|---|---|
| *Genotyping* | |
| SP0535-Forward | AATGAAAGCCATACGGGAAGCA |
| SP0535-Reverse | GCACACACTGACCAGATAACACAAAC |
| Cre-Forward | TCGATGCAACGAGTGATGAG |
| Cre-Reverse | TCCATGAGTGAACGAACCTG |
| *RT-qPCR* | |
| SP0535-Forward | GACTTGCCTGGAAGCAGACT |
| SP0535-Reverse | ATACGTGCCTCTGGGAGAGT |
| GAPDH-Forward (Mouse &Human) | CGGATTTGGTCGTATTGGG |
| GAPDH-Reverse (Mouse &Human) | CGCTCCTGGAAGATGGTGAT |
| Tubulin-Forward (Mouse &Human) | CCTACAACTCCATCCTCACC |
| Tubulin-Reverse (Mouse &Human) | ATCAAATCTCAGGGAAGCAG |
| Sox2-Forward (Mouse &Human) | CAAGATGCACAACTCGGAGA |
| Sox2-Reverse (Mouse &Human) | CGGGGCCGGTATTTATAATC |
| Pax6-Forward (Mouse &Human) | TCCATCAGTTTCCAACGGAGAA |
| Pax6-Reverse (Mouse &Human) | GTGGAATTGGTTGGTAGACAC |
| Eomes-Forward (Mouse &Human) | CACCGCCACCAAACTGAGAT |
| Eomes -Reverse (Mouse &Human) | CGAACACATTGTAGTGGGCAG |
| Bcl11b-Forward (Mouse) | CCCGACCCTGATCTACTCAC |
| Bcl11b -Reverse (Mouse) | CTCCTGCTTGGACAGATGCC |
| Bcl11b-Forward (Human) | ATCCTCAGCCCCTTTTGTTT |
| Bcl11b -Reverse (Human) | GCCGTTGTTCCTGAATTGTT |

| | |
|---|---|
| Satb2-Forward (Mouse) | GCCGTGGGAGGTTTGATGATT |
| Satb2-Reverse (Mouse) | ACCAAGACGAACTCAGCGTG |
| Satb2-Forward (Human) | GCAGTTGGACGGCTCTCTT |
| Satb2-Reverse (Human) | CACCTTCCCAGCTTGATTATTCC |
| Ki67-Forward (Mouse) | CGCAGGAAGACTCGCAGTTT |
| Ki67-Reverse (Mouse) | CTGAATCTGCTAATGTCGCCAA |
| Ki67-Forward (Human) | TTCGCAAGCGCATAACCCA |
| Ki67-Reverse (Human) | AACCGTGTCACAGTGCCAAA |
| DCX-Forward (Mouse) | GGCCAAGAAGGTACGTTTCTAC |
| DCX-Reverse (Mouse) | AGCAACGCATAAAACTACGAA |
| DCX-Forward (Human) | CAAGTCTAAGCAGTCTCTCCCATC |
| DCX-Reverse (Human) | ATAGCCCTGTTGGACACTTG |

**Table S4 Primers for Off-target analysis**

| Target | Sequence |
|---|---|
| gRNA1-OF1-Forward | GGGAGAGCTAAGACAGAGCA |
| gRNA1-OF1-Reverse | TCCCTCCTCCCCACCAAAAA |
| gRNA1-OF2-Forward | GCCTGCATAACTGTGTGATC |
| gRNA1-OF2-Reverse | GCAGGTTTTCAGCTACTCAC |
| gRNA1-OF3-Forward | ACTTACACCTCCACTGTCAG |
| gRNA1-OF3-Reverse | CACTGCTGCCCTGTATATGG |
| gRNA1-OF4-Forward | CTCTCTGTTCCTTCTGAGAG |
| gRNA1-OF4-Reverse | CCCCAATTCTATGCAGCTCT |
| gRNA1-OF5-Forward | CTCATGCAAATCCCGGACGT |
| gRNA1-OF5-Reverse | GCCGTGAACACTGAGAACGT |
| gRNA2-OF1-Forward | CCTTCTCTCTCACTGCAAGA |
| gRNA2-OF1-Reverse | GAGGATCCAGCATAAGACAG |
| gRNA2-OF2-Forward | GTTCATTCTTTACCGTGGTC |
| gRNA2-OF2-Reverse | GCTAGGTGTCTCTATGTGAT |
| gRNA2-OF3-Forward | CATGCTGAACAGGCTGAAGA |

| | |
|---|---|
| gRNA2-OF3-Reverse | GTGTCGTGTCTGTAACTACA |
| gRNA2-OF4-Forward | CATCCATCCTAAGCAGTGGC |
| gRNA2-OF4-Reverse | CACGAGGGTTTTCTGTGAGG |
| gRNA2-OF5-Forward | GGGAGCAGAACGTCTCTTCT |
| gRNA2-OF5-Reverse | GTCTTTTCTGCTCTCACCCC |

**Table S5 Plasmids information in HEK293T cell transfection**

| Description | Recombination Primer | Application |
|---|---|---|
| pCDH-CAG-IRES-mCherry | N/A | |
| pCDH-CAG-IRES-mCherry-SP0535 | Forward:<br>agggccgccactccaccggcggcatggacgagctgtacaagatgccactggaaaaattcgtagacatgg<br>Reverse:<br>atccagaggttgattgtcgacttaagcagtacatacgtgcctctggg | Figure S1C |
| pcDNA3.1(+)-CMV-SP0535-HA | Forward:<br>aaacttaagcttggtaccgagctcggatccatgccactggaaaaattcgtagacatggaa<br>Reverse:<br>gtaagcgtaatctggaacatcgtatgggtaagcagtacatacgtgcctctgggagagtct | Figure 6C, 6E-F |
| pcDNA3.1(+)-CMV-ATP1A1-Flag | Forward:<br>aaacttaagcttggtaccgagctcggatccatggggaagggggttggacgagacaagtat<br>Reverse:<br>gtaatccttatcgtcgtcatccttgtaatcgtagtaggtttccttctccacccagccgcca | Figure 6E-F |

mCherry sequence:

atggtgagcaagggcgaggaggataacatggccatcatcaaggagttcatgcgcttcaaggtgcacatggagggctccg
tgaacggccacgagttcgagatcgagggcgagggc
gagggccgcccctacgagggcacccagaccgccaagctgaaggtgaccaagggtggccccctgcccttcgcctggga
catcctgtcccctcagttcatgtacggctccaaggcctacgtgaagcaccccgccgacatccccgactacttgaagctgtcc
ttccccgagggcttcaagtgggagcgcgtgatgaacttcgaggacggcggcgtggtgaccgtgacccaggactcctccc
tgcaggacggcgagttcatctacaaggtgaagctgcgcggcaccaacttcccctccgacggccccgtaatgcagaagaa
gaccatgggctgggaggcctcctccgagcggatgtaccccgaggacggcgccctgaagggcgagatcaagcagaggc
tgaagctgaaggacggcggccactacgacgctgaggtcaagaccacctacaaggccaagaagcccgtgcagctgccc
ggcgcctacaacgtcaacatcaagttggacatcacctcccacaacgaggactacaccatcgtggaacagtacgaacgcg
ccgagggccgccactccaccggcggcatggacgagctgtacaagtga

HA sequence:

tacccatacgatgttccagattacgcttacccatacgatgttccagattacgcttacccatacgatgttccagattacgct

Flag sequence:

gattacaaggatgacgacgataaggattacaaggatgacgacgataaggattacaaggatgacgacgataag

**Table S6 Specific protein pulled by SP0535-HA in MS analysis**

| Gene name | MW (KDa) | Sequence Coverage (%) | # Unique Peptides | # Peptides | # PSMs | Abundances | Protein Score |
|---|---|---|---|---|---|---|---|
| Atp1a1 | 112.9 | 4 | 2 | 2 | 2 | 2531633.00 | 151 |
| My16b | 22.7 | 6 | 1 | 1 | 1 | 1881179.00 | 53 |
| Slc25a3 | 35.2 | 4 | 1 | 1 | 1 | 4180794.00 | 53 |
| Basp1 | 22.1 | 6 | 1 | 1 | 1 | 712359.50 | 39 |
| Ralgapa2 | 210.2 | 0 | 1 | 1 | 1 | 2035507.88 | 30 |
| Arhgef2 | 111.9 | 1 | 1 | 1 | 1 | 1426030.50 | 28 |

PSM：Peptide-spectrum matches.

**Table S7 Statistics and Quantification**

All statistical comparisons were conducted using the unpaired two-tailed Student's *t*-test.

| Figure | Number of Subjects | P value |
|---|---|---|
| 1e | All conditions=3 | $p$=0.0084 |
| 2a | All conditions=3 | $p$<0.0001 |
| 2c | All conditions=7 | $p$=0.0449 |
| 2f | All conditions=9 | $p$<0.0001 |
| 2g | All conditions=7 | $p$=0.0034 |
| 2i | All conditions=5 | $p$=0.0494 |
| 3e | All conditions=4 | $p$=0.0751 |
| 3f | All conditions=4 | $p$=0.0105 |
| 3g | All conditions=3 | 1: $p$=0.0015, 2: $p$=0.0011, 3: $p$=0.1210, 4: $p$=0.0132, 5: $p$=0.9545, 6: $p$=0.3001, 7: $p$=0.0747 |
| 4b | All conditions=5 | $p$=0.0198 |
| 4c | All conditions=5 | $p$=0.0450 |
| 4d | All conditions=3 | SOX2: $p$<0.0001, PAX6: $p$<0.0001 |
| 4f | All conditions=5 | $p$=0.0014 |
| 4g | All conditions=5 | $p$=0.0096 |
| 4h | All conditions=5 | $p$=0.0310 |
| 4i | All conditions=3 | TBR2: $p$=0.0010, CTIP2: $p$<0.0001, SATB2: $p$=0.0101 |
| 5b | All conditions=10 | $p$=0.0004 |
| 5c | All conditions=10 | $p$=0.0297 |
| 5e | All conditions=9 | $p$=0.0326 |

| | | |
|---|---|---|
| 5g | Learning: All conditions=15<br>Reversal: WT=7, TG=8 | D1: $p=0.6481$, D2: $p=0.3211$,<br>D3: $p=0.5507$, D4: $p=0.7279$,<br>D6: $p=0.5427$, D7: $p=0.2981$ |
| 5h | All conditions=15 | $p=0.0203$ |
| 5i | WT=7, TG=8 | $p=0.0088$ |
| 5k | All conditions=7 | D1: $p=0.2416$, D2: $p=0.0931$,<br>D3: $p=0.0889$, D4: $p=0.1860$,<br>D5: $p=0.0380$ |
| 5n | WT=110, TG=140 | $p<0.0001$ |
| 7b | All conditions=5 | $p=0.0390$ |
| 7d | All conditions=3 | $p<0.0001$ |
| 7e | All conditions=3 | $p=0.0116$ |
| 7f | All conditions=3 | ATP1A1: $p=0.0074$,<br>ATP1A1+SP0535: $p=0.0401$ |
| S2b | All conditions=3 | H9: D30: $p<0.0001$, D70: $p=0.2328$,<br>D100: $p=0.0362$; H1: D30: $p<0.0001$,<br>D120: $p<0.0001$ |
| S2e | All conditions=3 | $p=0.1304$ |
| S2f | All conditions=3 | $p=0.2917$ |
| S2h | All conditions=5 | $p=0.9339$ |
| S2j | All conditions=5 | $p=0.0004$ |
| S2k | All conditions=3 | $p=0.0008$ |
| S2l | All conditions=3 | $p=0.0019$ |
| S2m | All conditions=3 | $p=0.0001$ |
| S2n | All conditions=3 | $p=0.0007$ |
| S2o | All conditions=3 | $p<0.0001$ |
| S3b | All conditions=8 | $p=0.2868$ |
| S3e | All conditions=6 | $p=0.0058$ |
| S4b | All conditions=3 | $p=0.0392$ |

| | | |
|---|---|---|
| S4c | All conditions=5 | VZ: *p*=0.0403, SVZ: *p*=0.7112 |
| S4d | All conditions=3 | *p*=0.0028 |
| S4e | All conditions=3 | *p*=0.0128 |
| S4f | All conditions=3 | *p*=0.0261 |
| S4g | All conditions=3 | *p*=0.0013 |
| S4h | All conditions=3 | *p*<0.0001 |
| S4i | All conditions=3 | *p*=0.0004 |
| S5a | WT=8, TG=6 | *p*=0.7778 |
| S5b | All conditions=10 | *p*=0.6632 |
| S5c | All conditions=10 | *p*=0.0552 |
| S5d | All conditions=3 | *p*=0.6753 |
| S5f | All conditions=5 | *p*=0.6867 |
| S5h | All conditions=5 | *p*=0.6269 |
| S5i | All conditions=5 | *p*=0.3907 |
| S5j | All conditions=3 | *p*=0.0363 |
| S5m | All conditions=3 | *p*=0.7112 |
| S6d | All conditions=5 | *p*=0.0375 |
| S6f | All conditions=3 | ATP1A1: *p*=0.9255, Src: *p*=0.2125 |
| S6g | All conditions=3 | ATP1A1: *p*=0.2952, Src: *p*=0.4742 |
| S6h | All conditions=3 | ATP1A1: NC-ATP1A1: *p*=0.0201,<br>NC-ATP1A1+SP0535: *p*=0.0051;<br>ATP1A1-ATP1A1+SP0535: 0.3813<br>Src: NC-ATP1A1: *p*=0.4610,<br>NC-ATP1A1+SP0535: *p*=0.0993<br>ATP1A1-ATP1A1+SP0535: 0.4936 |
